# CD206^+^ tumor-associated macrophages cross-present tumor antigen and drive antitumor immunity

**DOI:** 10.1172/jci.insight.155022

**Published:** 2022-06-08

**Authors:** Madhura Modak, Ann-Kathrin Mattes, Daniela Reiss, Wioletta Skronska-Wasek, Rebecca Langlois, Nicolas Sabarth, Renate Konopitzky, Fidel Ramirez, Katharina Lehr, Tobias Mayr, David Kind, Coralie Viollet, Lee Kim Swee, Jutta Petschenka, Karim C. El Kasmi, Elfriede Noessner, Kerstin Kitt, Stefan Pflanz

**Affiliations:** 1 Department of Cancer Immunology and Immune Modulation, Boehringer Ingelheim Pharma, Biberach, Germany.; 2 Department of Biotherapeutics Discovery and; 3 Department of Cancer Immunology and Immune Modulation, Boehringer Ingelheim RCV GmbH & Co KG., Vienna, Austria.; 4 Department of Global Computational Biology and Digital Sciences and; 5 Department of Immunology and Respiratory, Boehringer Ingelheim Pharma, Biberach, Germany.; 6 Immunoanalytics Research Group Tissue Control of Immunocytes, Deutsches Forschungszentrum für Gesundheit und Umwelt, Helmholtz Zentrum München, Munich, Germany.

**Keywords:** Immunology, Oncology, Antigen presentation, Cancer immunotherapy, Macrophages

## Abstract

In many solid cancers, tumor-associated macrophages (TAM) represent the predominant myeloid cell population. Antigen (Ag) cross-presentation leading to tumor Ag–directed cytotoxic CD8^+^ T cell responses is crucial for antitumor immunity. However, the role of recruited monocyte-derived macrophages, including TAM, as potential cross-presenting cells is not well understood. Here, we show that primary human as well as mouse CD206^+^ macrophages are effective in functional cross-presentation of soluble self-Ag and non–self-Ag, including tumor-associated Ag (TAA), as well as viral Ag. To confirm the presence of cross-presenting TAM in vivo, we performed phenotypic and functional analysis of TAM from B16-F10 and CT26 syngeneic tumor models and have identified CD11b+F4/80hiCD206+ TAM to effectively cross-present TAA. We show that CD11b+CD206+ TAM represent the dominant tumor-infiltrating myeloid cell population, expressing a unique cell surface repertoire, promoting Ag cross-presentation and Ag-specific CD8+ T cell activation comparable with cross-presenting CLEC9A+ DCs (cDC1). The presence of cross-presenting CD206+ TAM is associated with reduced tumor burden in mouse syngeneic tumor models and with improved overall survival in cutaneous melanoma patients. Therefore, the demonstration of effective Ag cross-presentation capabilities of CD206+ TAM, including their clinical relevance, expands our understanding of TAM phenotypic diversity and functional versatility.

## Graphical abstract

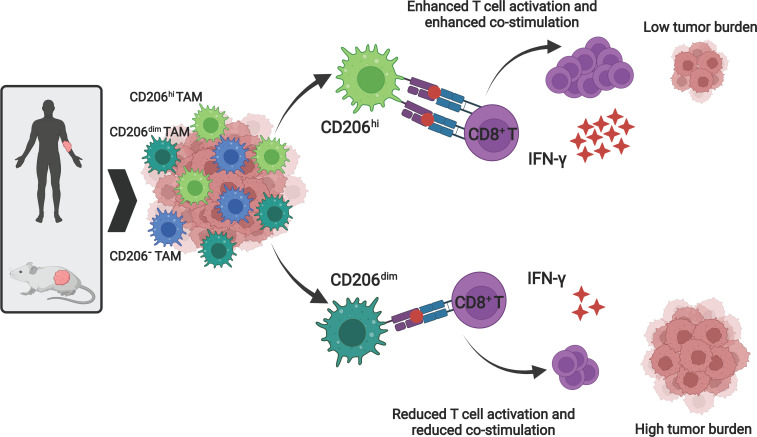

## Introduction

Achieving robust and specific CD8^+^ T cell responses is crucial for an effective antitumor immunity. Priming and activation of CD8^+^ T cells requires antigen (Ag) presentation in a complex with major histocompatibility class I (MHC I) by professional Ag-presenting cells (APC) (1). Ag cross-presentation enables the delivery of tumor-associated Ag (TAA) to the MHC I. Thus, efficient TAA cross-presentation by APC is a key requirement for the induction of an effective CD8^+^ T cell response against tumors.

Macrophages are known as vital sentinels of the immune system, with excellent phagocytic capacity. Embryonically derived tissue-resident macrophages, including lung alveolar macrophages and splenic as well as lymph node resident macrophages, are ideally positioned for Ag uptake (2). Additionally, various external cues can trigger extravasation and differentiation of circulating CD14^+^ monocytes to heterogenous macrophage subset populations that can be crudely characterized as classically activated, proinflammatory macrophages (M1) or alternatively activated, prorepair macrophages (M2) (3). Similarly, tumor-associated macrophages (TAM) represent the largest fraction of myeloid cell-infiltrates that differentiate from circulating monocytes and exhibit a continuum of macrophage subset phenotypes that can be characterized by the expression of cell surface markers including CD68, CD163, and CD206 (4, 5). So far, the M1 macrophages have been studied predominantly for their tumoricidal properties elicited by secretion of proinflammatory cytokines, including TNF, and activation of a Th1 response (6). In contrast, the role of M2 macrophages has mainly been studied in the context of angiogenesis, metastasis, and tumor progression mediated by the secreted growth factors (7). To date, the therapeutic strategies involving TAM in the solid tumor setting are mainly focused on depletion of TAM or on blocking of TAM recruitment and reprogramming of immunosuppressive M2-like TAM to M1-like macrophages (8, 9). Despite well-established phagocytic capacity of TAM, very little information is available on the possible role of TAM as cross-presenting APC and, hence, in the initiation and modulation of tumor Ag–specific CD8^+^ T cell responses by TAM subsets.

In the present study, we have identified Ag cross-presenting abilities of CD206^+^ macrophages in human as well as in the mouse syngeneic tumor models. Using a series of functional assays, we have shown that CD206^+^ macrophages were efficient in Ag uptake and cross-presentation of soluble Ag. CD206^+^ macrophages could effectively stimulate Ag-specific CD8^+^ T cells that subsequently acquired Ag-specific cytotoxic function. Furthermore, our data indicate that CD206^+^ macrophages were equipped with high expression of costimulatory receptors such as CD86 and ICOSLG, along with C-type lectin receptors such as CLEC4A that are key players in Ag delivery to the relevant cross-presentation pathways (10–12). In line with demonstrated Ag cross-presenting ability and unique cell surface repertoire expressed by CD206^+^ macrophages, increased frequency of CD11b^+^F4/80^hi^CD206^hi^ TAM was associated with enhanced Ag-specific CD8^+^ T cell activation and with concurrently reduced tumor burden in B16-F10 and CT26 syngeneic tumor models.

Our data provide unprecedented evidence to suggest that CD206^+^ macrophages, including TAM, should be considered as effective cross-presenting APC from a functional perspective, as well as by phenotypic marker characteristics. This has important implications for our perspective on TAM subset biology, including for the design of cancer immunotherapy strategies.

## Results

### CD206 expression positively correlates with overall survival of melanoma patients, and CD206^+^ TAM efficiently cross-present soluble TAA.

**Figure 1:**
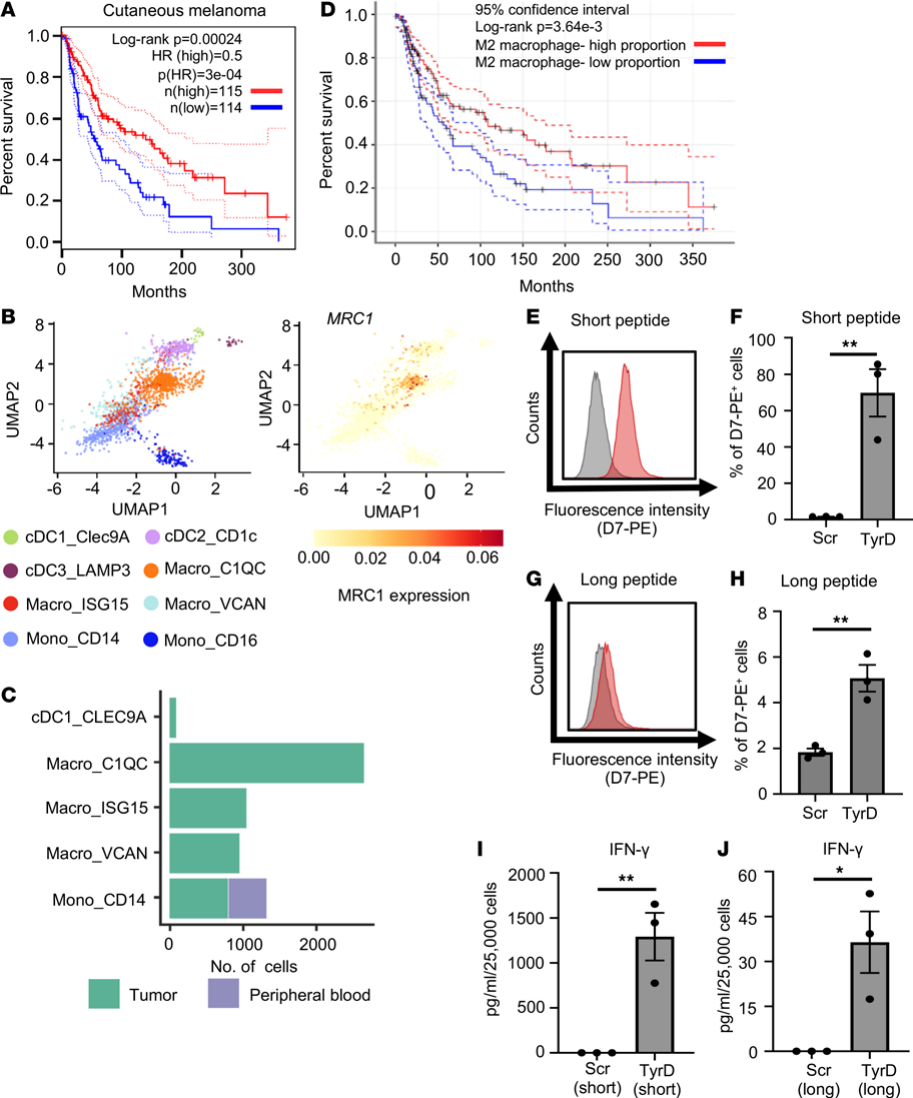
CD206^+^ TAM are detected in human melanoma tumors and can cross-present soluble TAA in vitro. (**A**) Overall survival analysis based on *MRC1* expression (blue, low *MRC1* TPM; red, high *MRC1* TPM) in cutaneous melanoma patients (*n* = 229) from the TCGA database, performed using GEPIA. (**B**) UMAP plots showing myeloid cell populations in melanoma colored by clusters (left) and expression of *MRC1* (CD206) for cell type annotation (right). (**C**) Bar diagram represents number of cells per indicated subsets between tumor tissue (green) and peripheral blood (violet) in melanoma patients. (**B** and **C**) The data are generated using scDVA tool. (**D**) Overall survival analysis based on proportion of M2 macrophage in tumor tissue (blue, low M2 macrophage proportion; red, high M2 macrophage proportion) in cutaneous melanoma patients (*n* = 469) from TCGA database. (**A** and **D**) Long-rank test. (**E**) Detection of TyrD_369–377_/MHC I complex with D7 TCRL antibody on in vitro–generated TAM. TAM were pretreated with TyrD short peptide (red histogram) or Scr short peptide (gray histograms). Data are representatives of 3 donors. (**F**) Bar diagram represents percentage of positive cells, as detected by reactivity profile of D7 antibody upon treatment of in vitro–generated TAM with short Scr peptide or TyrD peptide. (**G**) As in **E**, but cells were treated with Scr long peptide (gray histogram) or TyrD long peptide (red histogram). (**H**) As in **F**, but cells were treated with long Scr or TyrD peptide. (**I** and **J**) Bar diagrams represent IFN-γ levels in the T58/TAM coculture supernatant, where in vitro–generated TAM were pretreated with short Scr or TyrD peptide (**I**) and where in vitro–generated TAM were pretreated with long Scr or TyrD peptide (**J**). (**F** and **H**–**J**) Data show mean ± SEM (*n* = 3 donors). Unpaired Student’ *t* test was used. *P < 0.05, **P < 0.01.

In the tumor microenvironment, the presence of M2-like TAM, usually defined by the expression of CD163 and CD206, is generally regarded to exert immunosuppressive or proangiogenic effects (8, 9, 13, 14). However, a recent study has suggested that the presence of CD206^+^ macrophages correlated with improved survival in breast cancer patients (15). Similarly, we investigated the relationship between *MRC1* (also known as CD206) expression with clinical outcome data from the TCGA. We observed that higher expression of *MRC1* was associated with significantly better overall survival in cutaneous melanoma patients (Figure 1A). A similar association between high *MRC1* expression and better overall survival was also observed in renal cell carcinoma patients (Supplemental Figure 1A; supplemental material available online with this article; https://doi.org/10.1172/jci.insight.155022DS1). A detailed analysis of tumor-associated myeloid cells at single-cell resolution suggested that *MRC1* is specifically expressed by a TAM subset identified as Macro_C1QC (Figure 1, B and C, and Supplemental Figure 1B). We further confirmed a significant positive correlation between *MRC1* and *C1QC* expression in skin cutaneous melanoma patients from the TCGA database (Supplemental Figure 1C). Of note, higher expression of *C1QC* was also associated with better overall survival in these patients (Supplemental Figure 1D). Likewise, the presence of a higher proportion of M2 macrophages among immune cell-infiltrates was associated with improved overall survival in melanoma patients (Figure 1D), whereas a higher proportion of activated DCs among immune cell infiltrates did not correlate with improved overall survival in melanoma patients (Supplemental Figure 1E).

TAA cross-presentation is a crucial step in priming and activation of CD8^+^ T cells to initiate an effective antitumor immune response. Given the data described above, we hypothesized that TAM could be involved in TAA cross-presentation and analyzed the ability of TAM to cross-present TAA using melanoma-associated tyrosinase (TyrD) Ag as a model Ag. For this, as a source of Ag, we used a 27–amino acid TyrD-derived peptide that serves as a surrogate for TyrD protein. Long TyrD-derived peptide, similar to Ag protein, requires internalization and intracellular processing for possible Ag cross-presentation. The long TyrD peptide includes an epitope recognized by TyrD-specific TCRL D7 antibody, as well TyrD-specific T58-CD8^+^ T cells (16, 17). We used in vitro–generated human CD206^+^ TAM that faithfully recapitulate many characteristics of patient-derived TAM as recently described (18). Similar to previous reports, our in vitro–generated TAM expressed particularly high levels of CD206 and relatively low levels of CD80, as well as CD163 receptors (Supplemental Figure 2A). As a readout for cross-presentation, we leveraged the TCRL D7 antibody that can specifically detect the TyrD_369–377_/HLA-A02 complex but not the complex of other HLA-A02 restricted peptides or control scrambled (Scr) peptides on the cell surface (data not shown; Supplemental Figure 2, B–D) (16). Our data with TAP^–^ T2 cells show that the D7 antibody specifically detects cell surface presence of the TyrD_369–377_/HLA-A02 complex with short TyrD peptide but not with unprocessed long TyrD or Scr peptide (Supplemental Figure 2, B–D). Similarly, using TCRL D7 antibody, we could detect the presence of the TyrD_369–377_/HLA-A02 complex on the cell surface of about 70% of TAM upon treatment with TyrD short peptide but not with Scr short peptide (Figure 1, E and F). Furthermore, to assess cross-presentation of TyrD Ag, we analyzed the cells for the presence of cell surface TyrD_369–377_/HLA-A02 complex after incubating cells with long TyrD or Scr peptide. We detected the presence of the TyrD_369–377_/HLA-A02 complex on the TAM surface upon treatment with TyrD long peptide (Figure 1, G and H), confirming Ag cross-presentation capabilities of the TAM.

We next analyzed the ability of CD206^+^ TAM to stimulate TyrD-specific CD8^+^ T cells. For this, we used the human T58-CD8^+^ T cells that express a high-avidity TyrD_369–377_/HLA-A02–specific T cell receptor (17). Marking of the TAM with the short TyrD peptide but not with the short Scr peptide, prior to coculture, led to secretion of IFN-γ, as expected (Figure 1I). Next, we assessed T cell stimulation capacity following cross-presentation. TAM were incubated with long TyrD peptide or control Scr peptide and then were cocultured with T58 cells. Consistent with the findings with the D7 TCRL antibody, following cross-presentation of long TyrD peptide, TAM could also activate TyrD-specific T58 cells in an Ag-specific manner (Figure 1J).

### Delayed tumor progression is linked to increase in tumor-associated CD11b^+^F4/80^hi^CD206^+^ cross-presenting TAM and enhanced Ag-specific CD8^+^ T cell activation.

Analysis of TCGA data set suggests an association of higher CD206 expression, as well the presence of M2 macrophages with better clinical prognosis in patients with melanoma and renal cell carcinoma (Figure 1). Corroborating in silico results, data from in vitro generated CD206^+^ TAM suggested that human CD206^+^ TAM are capable of cross-presenting soluble TAA, in the form of long Ag peptide, and that — following Ag cross-presentation — TAM can effectively stimulate Ag-specific CD8^+^ T cells (Figure 1J). To further confirm these findings in an in vivo setting, we opted for the widely used B16-F10 melanoma syngeneic tumor model (19).

**Figure 2:**
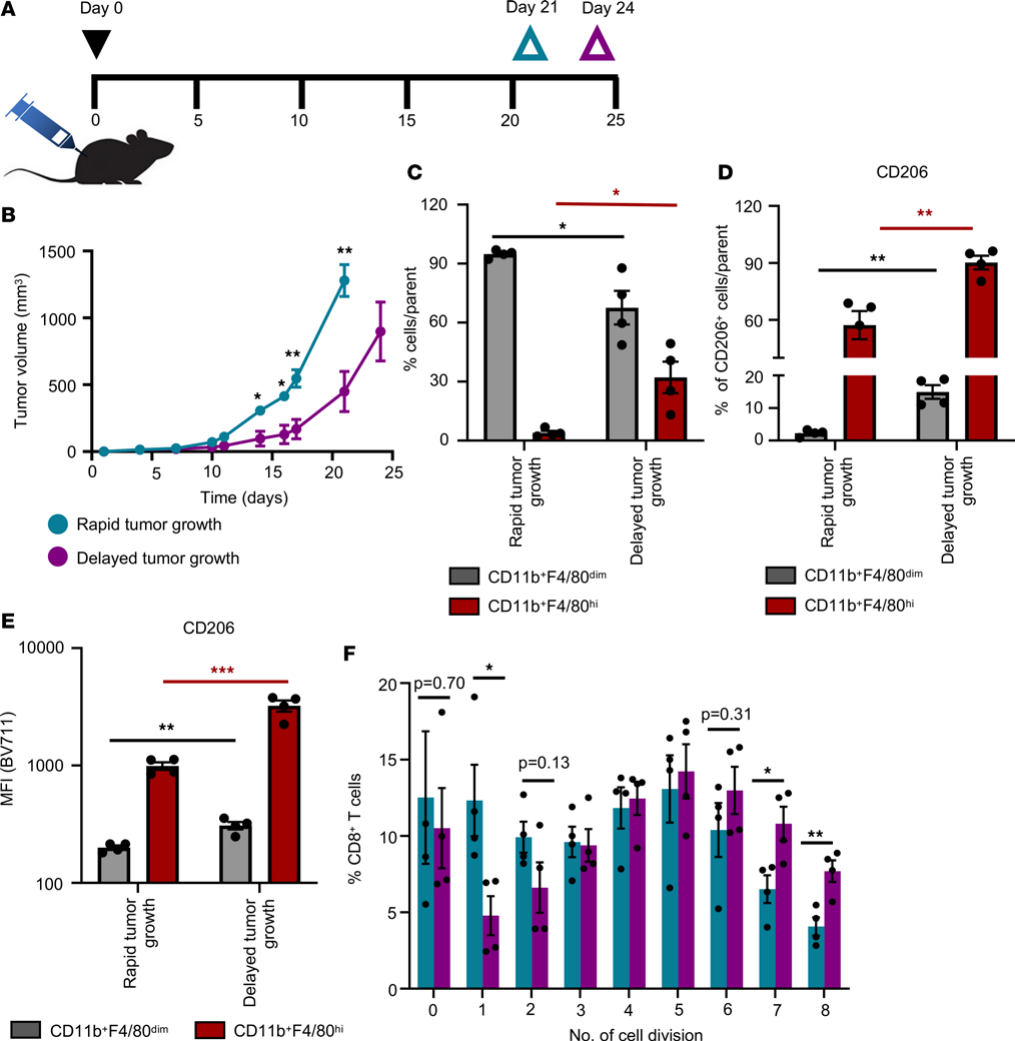
B16-F10 melanoma isolated CD206^+^ TAM are efficient in cross-presentation of soluble OVA Ag. (**A**) Timeline of the murine syngeneic tumor model studies, indicating inoculation of B16-F10 melanoma cells (day 0) and respective endpoints (day 21, rapid-growth tumors; day 24, delayed-growth tumors). (**B**) In vivo B16-F10 tumor growth curves by 2 groups identified as rapid-growth tumor (cyanine blue) and delayed-growth tumor (purple). (**C**) Bar diagram represents percentage of CD11b^+^F4/80^dim^ (gray) and CD11b^+^F4/80^hi^ (red) TAM analyzed in rapid- and delayed-growth tumors by flow cytometry. (**D** and **E**) Bar diagrams represent percentage (**D**) and mean fluorescence intensity (MFI) (**E**) of CD206^+^ cells from CD11b^+^F4/80^dim^ (gray) and CD11b^+^F4/80^hi^ (red) TAM analyzed in rapid- and delayed-growth tumors by flow cytometry. (**F**) Bar diagram represents percentage of CD8a^+^ T cells in indicated cell division peak upon coculture with TAM (CD11b^+^F4/80^+^) isolated from rapid-growth tumors (cyanine blue) or delayed-growth tumors (purple). (**B**–**F**) Data show mean ± SEM (*n*= 4 mice per group). Unpaired Student’s *t* test was used. For multiple comparisons Holm-Šídák test was performed (**C**–**E**). *P < 0.05, **P < 0.01, ***P < 0.001. Red asterisk indicate the statistical analysis of CD11b^+^F4/80^hi^ cells between rapid and delayed tumor growth. Black asterisks indicate the statistical analysis of CD11b^+^F4/80^dim^ cells between rapid and delayed tumor growth.

C57BL/6 mice bearing rapid-growth tumors were sacrificed at day 21 upon reaching the tumor size between 1000 mm^3^ and 1500 mm^3^. Day 24 was considered as an experimental endpoint, and mice bearing delayed-growth tumors were sacrificed on day 24 (Figure 2, A and B) (19). Mice showed significant differences in tumor growth between rapid-growth tumor and delayed-growth tumor groups from day 14 onwards (Figure 2B). We next isolated TAM from the excised tumors using flow cytometry-based cell sorting as described (Supplemental Figure 3A). Comprehensive analysis of TAM isolated from rapid- and delayed-growth tumors revealed that the TAM population contains 2 distinct subsets that can be defined as CD11b^+^F4/80^dim^ and CD11b^+^F4/80^hi^ (Supplemental Figure 3B). The percentage of CD11b^+^F4/80^hi^ TAM population from Lin^–^CD45^+^ cells was significantly more abundant in delayed-growth tumors, whereas rapid-growth tumors contained a higher percentage of CD11b^+^F4/80^dim^ TAM (Figure 2C). The significant differences in tumor growth kinetics between the 2 groups was likely not due to differences in monocyte recruitment. We did not see significantly increased CCL2 levels in plasma of mice with delayed-growth tumor compared with mice with rapid-growth tumor, even though mice with delayed-growth tumors had significantly elevated levels of CCL2 compared with tumor naive mice (Supplemental Figure 3D). Furthermore, comparative analysis of CD206 expression between CD11b^+^F4/80^dim^ and CD11b^+^F4/80^hi^ TAM among each group suggested that CD206 expression was significantly higher in CD11b^+^F4/80^hi^ compared with CD11b^+^F4/80^dim^ TAM (Supplemental Figure 3, B and C). In line with the results obtained with the TCGA dataset in melanoma patients, the percentage of CD206^+^ TAM was higher in delayed-growth tumors compared with rapid-growth tumors (Figure 2D). On a per-cell basis CD11b^+^F4/80^dim^ as well as CD11b^+^F4/80^hi^ TAM from delayed-growth tumors expressed significantly higher levels of CD206 compared with TAM isolated from rapid-growth tumors (Figure 2, D and E).

Of note, using the CT26 colon carcinoma model, we have analyzed the TAM population from rapid- and delayed-growth tumors. Similar to our findings in the B16-F10 melanoma model, a significantly higher percentage of the CD11b^+^F4/80^hi^ TAM population can be found in delayed-growth tumors compared with rapid-growth tumors (Supplemental Figure 3, E–G). Furthermore, in the CT26 colon carcinoma model, CD11b^+^F4/80^hi^ TAM from delayed-growth tumors were also characterized by significantly higher expression CD206, compared with that in rapid-growth tumors (Supplemental Figure 3H).

Next, the sorted CD11b^+^F4/80^+^ TAM from B16-F10 tumors were exposed to either OVA or BSA (control) soluble proteins and were analyzed for their ability to cross-present Ag and subsequently activate naive CD8a^+^ T cells isolated from tumor-free OT-I transgenic mice. TAM isolated from rapid- as well as delayed-growth tumors could stimulate the naive CD8^+^ T cells in an Ag-specific manner (Figure 2F and Supplemental Figure 4, A–C). The detailed analysis of CD8a^+^ T cells per cell division showed a significantly higher percentage of proliferating CD8a^+^ T cells in later cell division cycles (e.g., division cycles 7 and 8) when CD8a^+^ T cells were stimulated with OVA-exposed TAM isolated from delayed-growth tumors compared with when CD8a^+^ T cells were stimulated with OVA-exposed TAM from rapid-growth tumors. These data suggest that TAM from delayed-growth tumors are more potent in Ag cross-presentation and in inducing sustained CD8^+^ T cell proliferation compared with TAM from rapid-growth tumors (Figure 2F).

**Figure 3:**
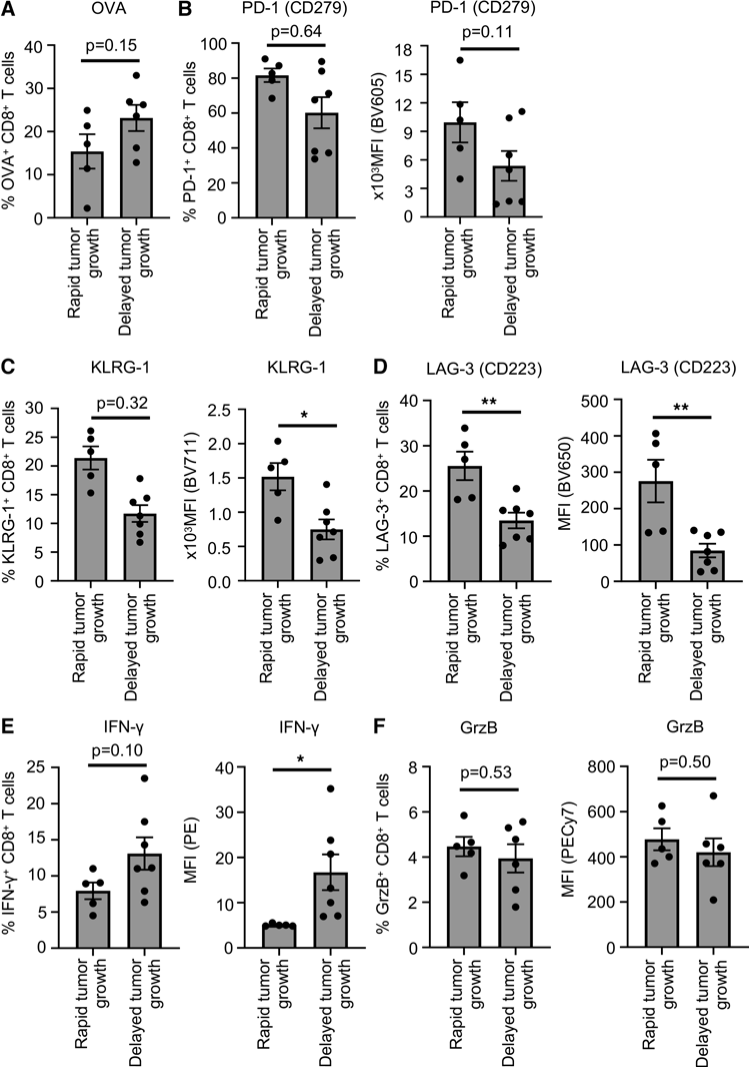
Characterization of tumor-associated CD8^+^ T cells from B16-F10 melanoma tumor. (**A**) Bar diagram represents percentage of OVA-specific TCR-expressing tumor-associated CD8^+^ T cells in rapid- and delayed-growth tumors by flow cytometry. (**B**–**F**) Bar diagrams show percentage of PD-1^+^CD8^+^ T cells (**B**), KLRG-1^+^CD8^+^ T cells (**C**), LAG-3^+^CD8^+^ T cells (**D**), IFN-γ^+^CD8^+^ T cells (**E**), and Granzyme B (GrzB)^+^CD8^+^ T cells (**F**) with corresponding MFI from rapid- and delayed-growth tumors as analyzed by flow cytometry. (**A**–**F**) Data show mean ± SEM (*n* = 5–7 mice per group). Unpaired Student’s *t* test was used. *P < 0.05, **P < 0.01.

Furthermore, to analyze the direct effect of abundance of Ag–cross-presenting CD11b^+^F480^hi^CD206^+^ TAM on CD8^+^ T cell activation in vivo, we have also assessed the tumor-associated CD8^+^ T cell population, comparing rapid- and delayed-growth tumors. Tumor-associated CD8^+^ T cells from delayed growth B16-F10–OVA tumors showed increased frequency of OVA-specific TCR-expressing CD8^+^ T cells compared with rapid-growth tumors (Figure 3A). Furthermore, CD8^+^ T cells from delayed-growth tumors expressed low levels of inhibitory receptors such as PD-1, KLRG-1, and LAG-3 compared with CD8^+^ T cells from rapid-growth tumors (Figure 3, B–D). As another readout for successful tumor Ag cross-presentation, delayed-growth tumors also showed high frequency of IFN-γ–expressing CD8^+^ T cells, compared with rapid-growth tumors (Figure 3, E and F). Additionally, we have also characterized the tumor-associated CD8^+^ T cells from the CT26 tumor model. The analysis of CD8^+^ T cells from CT26 also confirmed the enhanced Ag-specific activation of CD8^+^ T cells from delayed-growth tumors compared with rapid-growth tumors (Supplemental Figure 5). Together, these data suggest that high prevalence of CD11b^+^F4/80^hi^CD206^+^ Ag cross-presenting TAM correlates with enhanced Ag-specific activation of tumor-associated CD8^+^ T cells.

Thus, our data from the in vivo B16-F10 and CT26 murine tumor models are consistent with our findings using in vitro–generated CD206^+^ human TAM and with the expression profiles from melanoma patient specimens, described above, further confirming the capabilities of CD206^+^ TAM as a potent Ag–cross-presenting cell population in the tumor microenvironment.

### Alternatively activated CD206^+^ M2a macrophages efficiently cross-present soluble TAA.

Our data, so far, suggest that CD206^+^ TAM are efficient in cross-presentation of soluble Ag. Similar to TAM, in the setting of ongoing immune responses, circulating CD14^+^ monocytes can be recruited to the tissue, where they differentiate into diverse functional monocyte-derived macrophage (MDM) subsets. In the next set of experiments, we analyzed the Ag–cross-presenting ability of various MDM subsets. For this, CD14^+^ monocytes were differentiated to classically activated CD80^hi^CD163^lo^CD206^lo^ M1 macrophages and 2 subpopulations of alternatively activated M2 macrophages that can be distinguished based on CD206 expression as CD80^lo^CD163^lo^CD206^hi^ M2a macrophages and CD80^lo^CD163^hi^CD206^lo^ M2c macrophages by adding the indicated stimuli (Supplemental Figure 6, A–C).

**Figure 4:**
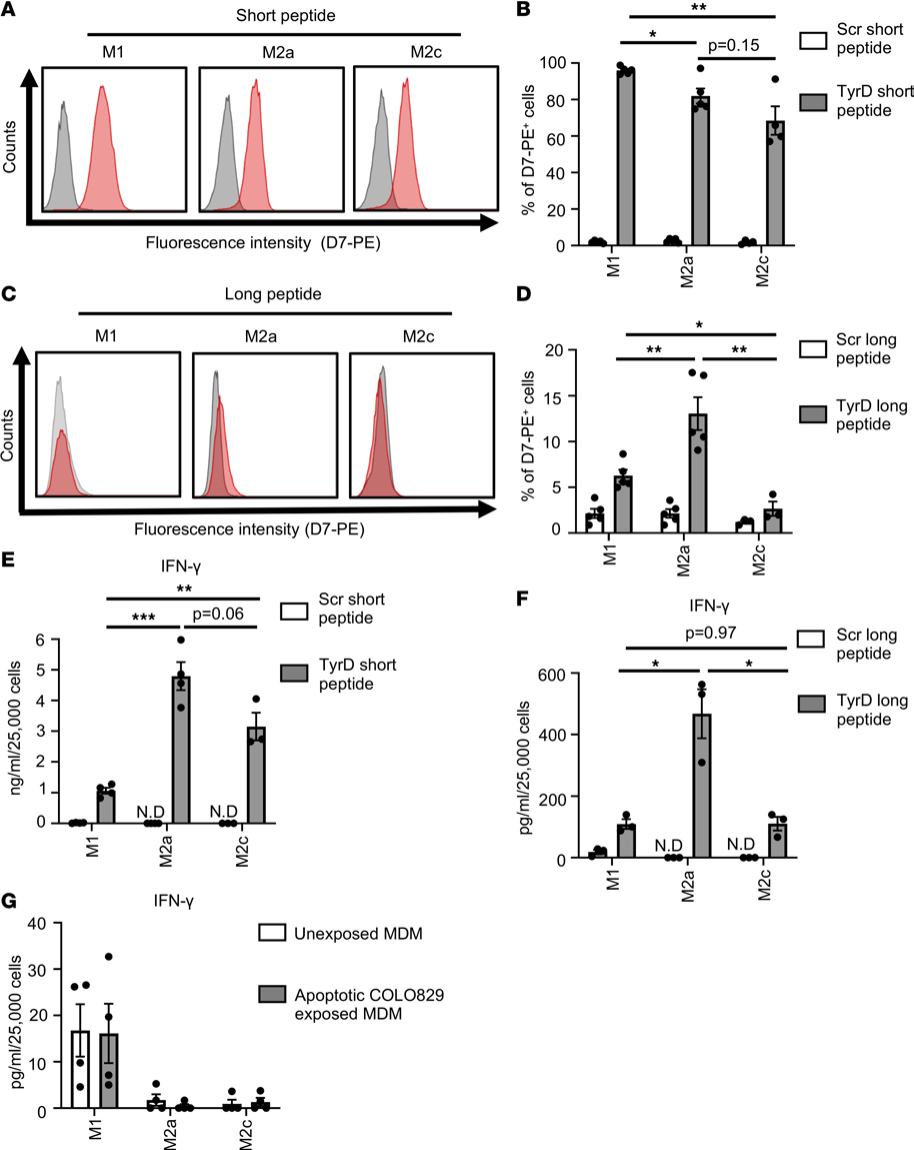
CD206^+^ macrophages are efficient in cross-presenting soluble TAA. (**A**) Detection of TyrD_369–377_/MHC I complex with D7 TCRL antibody on indicated MDM subsets. MDM subsets were pretreated with TyrD short peptide (red histogram) or Scr short peptide (gray histograms). (**B**) Bar diagram represents percentage of positive cells as detected by reactivity profile of D7 antibody upon treatment of MDM subsets with Scr short peptide (open bars) and TyrD short peptide (filled bars). (**C**) As in **A**, but MDM were treated with TyrD long peptide (red histogram) or with Scr long peptide (gray histogram). (**A** and **C**) Data are representative from 5 different donors. (**D**) As in **B**, but MDM were treated with Scr long peptide (open bars) or TyrD long peptide (filled bars). (**B** and **D**) Data show mean ± SEM (*n* = 4–5 donors). (**E** and **F**) Bar diagram represents IFN-γ levels in the T58/MDM coculture supernatant where MDM subsets were pretreated with Scr short peptide (open bars) or TyrD short peptide (filled bars) (**E**), and where MDM were pretreated with Scr long peptide (open bars) or TyrD long peptide (filled bars) (**F**). Data show mean ± SEM (*n* = 3–4 donors). (**G**) Bar diagram represents IFN-γ levels in the T58/MDM coculture supernatants as analyzed by IFN-γ CBA assay, wherein MDM were unexposed (open bars) or were first exposed to UV-treated apoptotic COLO829 cells for 5 hours (filled bars). Data show mean ± SEM (*n* = 4 donors). (**B** and **D**–**F**) Unpaired Student’s *t* test followed by Holm-Šídák test for multiple comparisons was performed. *P < 0.05, **P < 0.01, ***P < 0.001.

We then analyzed the Ag–cross-presenting ability of MDM using TyrD Ag. The presence of short TyrD peptide but not the Scr peptide in complex with HLA-A02 can be clearly detected with the TCRL D7 antibody on the surface of MDM (Figure 4, A and B). Furthermore, to assess cross-presentation of TyrD Ag, we analyzed the cells for the presence of cell surface TyrD_369–377_/HLA-A02 complex after incubating cells with long TyrD or Scr peptide. Based on D7 TCRL antibody staining, the CD206^+^ M2a macrophages showed significantly higher presence of the TyrD_369–377_/HLA-A02 complex on their surface (13% ± 3.9%) compared with donor-matched M1 (6.2% ± 1.4%) and M2c (2.6% ± 1.3%) macrophages (Figure 4, C and D). We further verified the detection of TyrD_369–377_/HLA-A02 with D7 antibody on cross-presenting CLEC9A^+^ human DCCs (cDC1) using TyrD short and long peptide (Supplemental Figure 7, A and B). Data with TyrD long peptide using CLEC9A^+^ cDC1 indicate that cell surface TyrD_369–377_/HLA-A02 complexes could be detected on 6.5% ± 1.2% of cells (Supplemental Figure 7B).

We next analyzed the ability of MDM subsets to stimulate TyrD-specific T58 cells. Our data show that MDM subsets — as well as CLEC9A^+^ cDC1 treated with short TyrD peptide but not with the short Scr peptide, prior to coculture — could stimulate T58 cells (Figure 4E and Supplemental Figure 7C). Next, MDM were incubated with long TyrD peptide or control Scr peptide and then were cocultured with T58 cells. Consistent with the findings with the D7 TCRL antibody, following effective cross-presentation, CD206^+^ M2a macrophages efficiently stimulated TyrD-specific T58 cells. The levels of IFN-γ in the supernatants were significantly higher with M2a macrophages (467 ± 79.9 pg/mL) pretreated with long TyrD peptide compared with donor-matched M1 (109 ± 15.6 pg/mL) or M2c (110 ± 22.3 pg/mL) macrophages (Figure 4F). Interestingly, in a corresponding experiment, using CLEC9A^+^ cDC1, the levels of IFN-γ in the supernatants were found to be 885 ± 280.4 pg/mL (Supplemental Figure 7D). Assessment of IFNγR1 (CD119) expression levels on MDM subsets suggested that the differential levels of IFN-γ detected in the supernatants were unlikely to result from IFNγR-dependent IFN-γ consumption by the MDM subsets (Supplemental Figure 7, E and F).

After establishing the role of CD206^+^ macrophages, including TAM, in Ag cross presentation of soluble Ag, we next analyzed whether these cells can effectively cross-present cell-derived Ag. For this, we used the HLA-A02^–^ melanoma cell line COLO829, for which TyrD protein expression has been reported (20). Induction of apoptosis in UV-treated COLO829 cells was confirmed 24 hours later using Annexin V/7-AAD via flow cytometry. To qualify for a use in the uptake experiment, more than 60% of the UV-treated COLO829 cells were confirmed to be annexin V^+^/7-AAD^+^ (data not shown). MDM from HLA-A02^+^ donors were cultured alone (control) or with apoptotic COLO829 cells for 5 hours. Subsequently, T58 cells were added to the culture (MDM/T58 ratio of 1:5), and activation of TyrD-specific T58 cells was analyzed. Of note, by choosing the HLA-A02^–^ COLO829 cell line, we aimed to ensure that any possible stimulation signal for TyrD-specific T58 cells could exclusively arise from the TyrD Ag cross-presented by MDM in this experimental setting. No difference in IFN-γ response by T58 cells was detectable in the comparison of control-incubated and COLO829-incubated MDM subsets in this assay (Figure 4G). The detected IFN-γ levels were in the range of about 15 pg/mL or below; therefore, they were just above the lower limit of quantification of the CBA assay, similar to those detected with Scr control–treated MDM subsets in other experiments (Figure 4, E–G). Consequently, our data suggest that cross-presentation of cell-derived TyrD Ag leading to cell surface TyrD_369–377_/HLA-A02 complexes was minimal or absent in the experimental setting of cell-associated TyrD Ag, unlike in the setting with TyrD Ag long peptide, described above.

### CD206^+^ macrophages effectively ingest Ag but process differentially to favor Ag cross-presentation.

**Figure 5:**
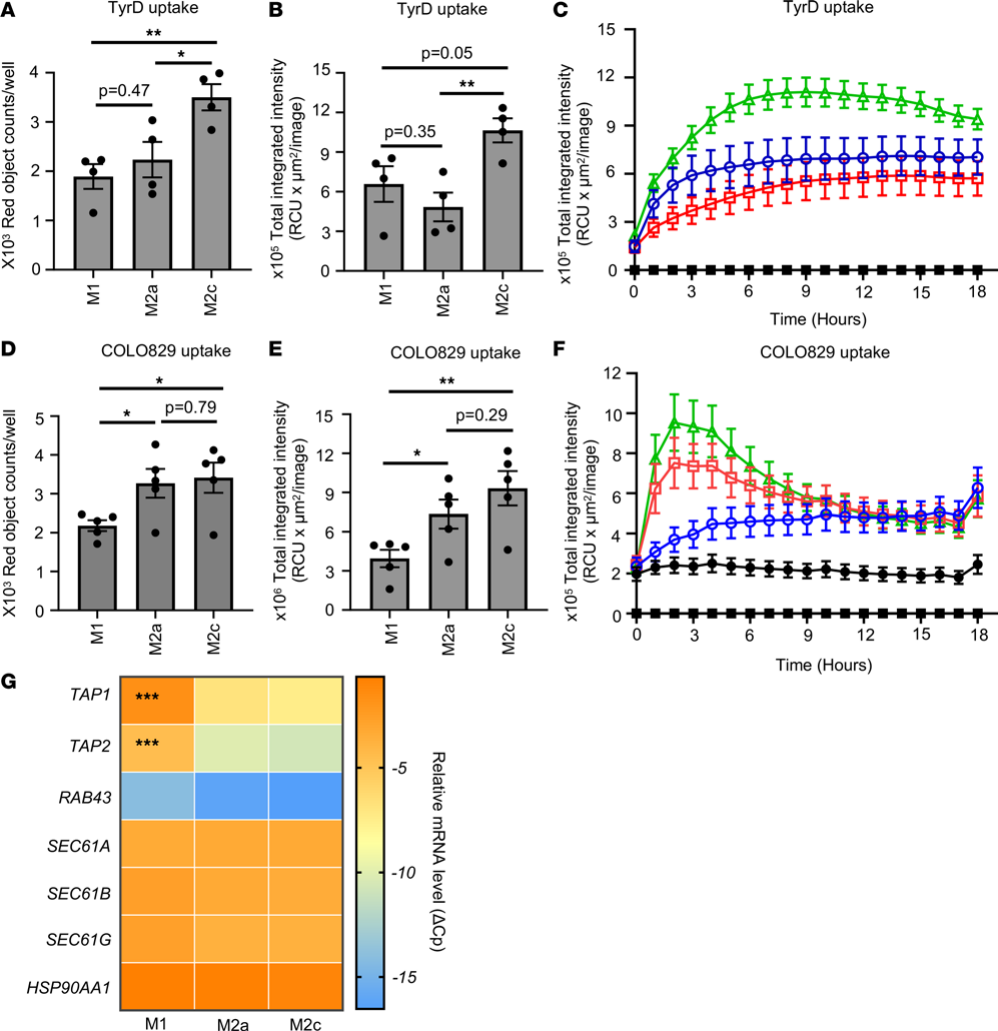
Ag uptake and processing by MDM subsets. (**A**–**C**) Differentiated human MDM were coincubated with pHrodo Red–labeled TyrD long peptide. Ag uptake was analyzed using Incucyte S3 live cell analysis system. (**A** and **B**) Bar diagrams represent number of red objects/well (**A**) and total integrated intensity (**B**) as analyzed at 6 hours. (**C**) M1 (blue circle), M2a (red square), and M2c (green triangle) MDM were coincubated with pHrodo Red–labeled TyrD long peptide. Ag uptake was recorded over 18 hours. The graph also shows the baseline values for peptide alone and MDM subsets alone (black squares). (**D**–**F**) Differentiated human MDM were coincubated with pHrodo Red–labeled UV-treated apoptotic COLO829 cells. Uptake of apoptotic cells was recorded using Incucyte S3 live cell analysis system. (**D** and **E**) Bar diagrams represent number of red objects/well (**D**) and total integrated intensity (**E**) as analyzed using Incucyte software at 3 hours. (**F**) Uptake of apoptotic cells by M1 (blue circles), M2a (red squares), and M2c (green triangles) MDM subsets was also recorded over 18 hours. The graph shows the baseline values for COLO829 alone (black circles) and MDM subsets alone (black squares). (**D**–**F**) Data show mean ± SEM (*n* = 5 donors). (**G**) Heatmap indicating mRNA expression of *TAP1*, *TAP2*, *RAB43*, *SEC61A*, *SEC61B*, *SEC61G*, and *HSP90AA1*. (**A**–**C** and **G**) Data show mean ± SEM (*n* = 4 donors). Unpaired Student’s *t* test was used. For multiple comparisons, Holm-Šídák test was performed (**A**, **B**, **D**, and **E**). *P < 0.05, **P < 0.01, ***P < 0.001.

Ag uptake is a required first step in the cascade, ultimately resulting in Ag cross-presentation. Therefore, we next analyzed the ability of MDM to uptake Ag in a soluble as well a cell-derived particulate form. The data show that all MDM subsets are capable of effective ingestion of the pHrodo Red–labeled TyrD long peptide, by absolute count of ingested red objects at the 6-hour time point (Figure 5A). Despite potent TyrD peptide uptake, M2a macrophages showed lower total integrated fluorescence intensity compared with M1 or M2c macrophages as analyzed at the 6-hour time point or continuously over 18 hours (Figure 5, B and C). Ag uptake capabilities by all 3 MDM subsets were further confirmed using an unrelated soluble Ag, dextran (Supplemental Figure 8, A–C).

Lack of detectable cross-presentation did not seem to occur due to inefficiency of MDM subsets to ingest apoptotic COLO829 cells. Our data with pHrodo Red–labeled apoptotic COLO829 cells show that, compared with M1 macrophages, both M2a and M2c macrophages were effectively ingesting the dying COLO829 cells (Figure 5D). Higher integrated intensities observed with M2a and M2c macrophages compared with M1 macrophages at the 3-hour time point suggest that, following ingestion, tumor cells were exposed to lysosomal acidic pH and, therefore, to more efficient degradation in M2a and M2c macrophages compared with M1 macrophages (Figure 5E). As analyzed over the period of 18 hours, both M2a and M2c macrophages showed higher total integrated intensities compared with M1 macrophages between 2 and 4 hours; these intensities then started to decline, reaching levels similar to M1 after 9 hours (Figure 5, E and F). This effect might result from initial faster uptake of apoptotic cells and subsequent lysosomal degradation of engulfed cells, resulting in lack of availability of target cells.

The observed differences in Ag uptake efficiency by MDM subsets did not result from variable cell densities, as illustrated by the percentage of confluence analyzed at 0 hours (Supplemental Figure 8D).

Our data with 2 independent soluble Ags of different molecular weight and different biochemical composition, as well as with cell-derived Ag, confirm that MDM, including CD206^+^ macrophages, can efficiently ingest Ag in different forms. However, upon ingestion, the soluble Ags might be processed differentially by MDM, resulting in different levels of Ag cross-presentation. We next analyzed the expression of molecular regulators of cross-presentation. All MDM subsets expressed similar levels of SEC61 subunits — a translocon for degraded peptides from endosome to cytosol as well as of HSP90 subunit *HSP90AA1* — a chaperon involved in cytosolic translocation of processed peptides (Figure 5G) (21, 22). Similarly, all MDM subsets expressed *TAP1* and *TAP2* subunits of the TAP heterodimer. However, alternatively activated M2a and M2c macrophages seem to express low levels of *TAP1* and *TAP2* compared with classically activated M1 macrophages (Figure 5G). RAB43 is a small GTPase that has been shown to be important for cross-presentation of cell-derived Ag by CD8α^+^ DCs (23). All MDM subsets expressed negligible levels of *RAB43*, as analyzed by quantitative PCR (qPCR) (Figure 5G). These data might explain the ability of MDM to effectively cross-present TAA in soluble long Ag peptide form, less so in cell-derived form.

### CD206^+^ macrophage-stimulated Ag-specific CD8^+^ T cells acquire potent cytotoxic capacity.

**Figure 6:**
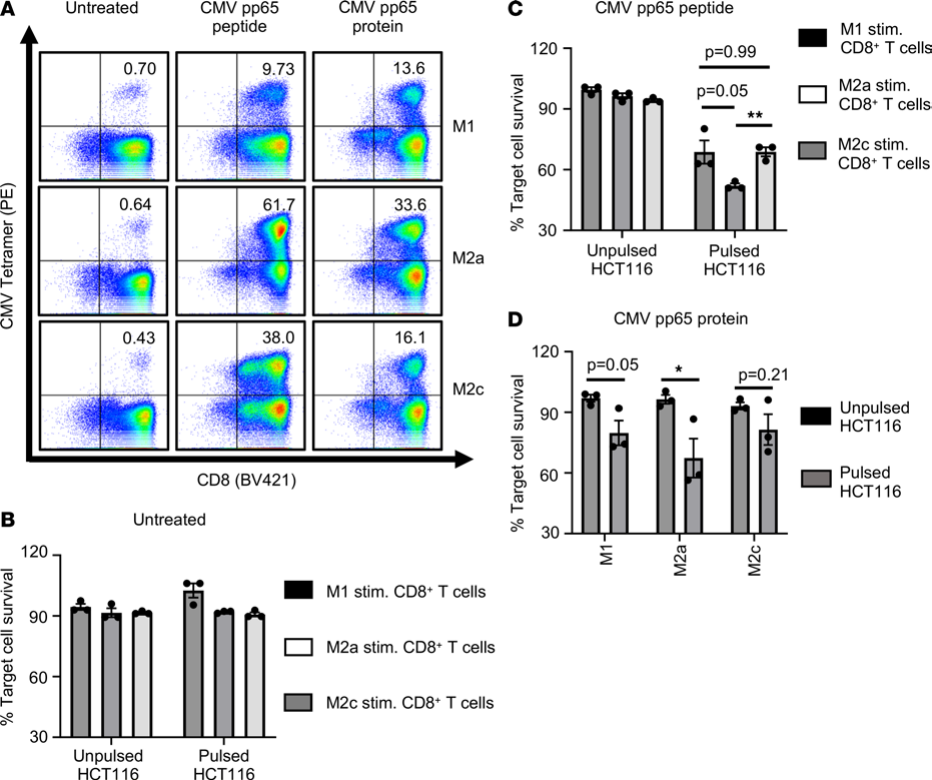
CD206^+^ macrophages can efficiently cross-present soluble CMV pp65 Ag. (**A**) Dot plots indicate expression of CMV-specific TCR on CD8^+^ T cells as analyzed by MHC I tetramer staining. CD8^+^ T cells were stimulated with untreated MDM or MDM pretreated with CMV pp65 short peptide, or they were stimulated with MDM pretreated with CMV pp65 protein. Numbers indicate percentage of CD8^+^CMV^+^ T cells. Data are representative of 3 donors. (**B**–**D**) Data show cytotoxic activity of differentially stimulated CD8^+^ T cells, wherein autologous CD8^+^ T cells were cocultured with untreated (**B**), CMV pp65 peptide pretreated (**C**), or CMV pp65 protein pretreated (**D**) MDM subsets. Bar diagrams show percentage survival of target HCT116 cells, cocultured with differentially stimulated CD8^+^CMV^+^ T cells, normalized to HCT116 cells alone. HCT116 cells were either unpulsed or pulsed with CMV pp65 peptide before coculturing with stimulated CD8^+^ T cells. (**B**–**D**) Data show mean ± SEM (*n* = 3 donors). Unpaired Student’s *t* test was used. For multiple comparisons, Holm-Šídák test was performed (**C**). *P < 0.05, **P < 0.01.

We next aimed to verify the cross-presenting ability of CD206^+^ macrophages using a completely unrelated soluble Ag, recombinant CMV pp65 protein. MDM were either left untreated or pretreated with CMV pp65 peptide or recombinant CMV pp65 protein, as indicated. Isolated autologous primary human CD8^+^ T cells were then cocultured with either untreated or Ag-stimulated MDM, as described earlier (24). Upon Ag-specific activation and expansion, CD8^+^ T cells were analyzed for the expression of CMV-specific TCR by MHC I tetramer staining. Untreated MDM were used as control and did not induce expansion of CMV-specific CD8^+^ T cells, as anticipated (Figure 6A). Nonspecific tetramer was used as a negative control and showed no differential staining, as expected (data not shown). CMV protein–challenged MDM-stimulated CD8^+^ T cells showed a clear increase in Ag-specific CD8^+^ T cells compared with control untreated MDM (Figure 6A). Thus, our results show that MDM can effectively cross-present CMV Ag and can subsequently cross-prime and stimulate Ag-specific CD8^+^ T cells.

Next, we analyzed whether MDM-stimulated Ag-specific CD8^+^ T cells acquire cytotoxic activity. To analyze Ag-specific cytotoxicity of CD8^+^ T cells, we used HCT116 cells that were either left untreated or pulsed with CMV peptide. Our data show that only Ag-expanded CD8^+^ T cells showed CMV-specific cytotoxicity, specifically killing CMV pp65 peptide-pulsed HCT116 cells but not unpulsed target cells (Figure 6, B–D). Furthermore, Ag-specific CD8^+^ T cells stimulated by CD206^+^ macrophages showed significantly enhanced Ag-specific cytotoxic activity compared with M1- or M2c-stimulated Ag-specific CD8^+^ T cells (Figure 6, C and D).

Thus, our data confirm the cross-presenting capabilities of CD206^+^ macrophages with a completely unrelated non–self-Ag and further demonstrate that, following cross-presentation, CD206^+^ macrophage-stimulated Ag-specific CD8^+^ T cells elicit potent cytotoxic function.

### CD206^+^ macrophages express high levels of costimulatory receptors CD86 and ICOSLG.

We have shown that CD206^+^ macrophages, including TAM and M2a MDM, are capable of not only cross-presenting soluble Ag, but also efficiently stimulating Ag-specific CD8^+^ T cells (Figure 1, Figure 2, Figure 3, Figure 4, and Figure 6). Therefore, using single-cell RNA-Seq (scRNA-Seq), we analyzed the expression of various costimulatory and coinhibitory receptors on MDM subsets and further confirmed expression of key cell surface receptors on MDM subsets via flow cytometry.

**Figure 7:**
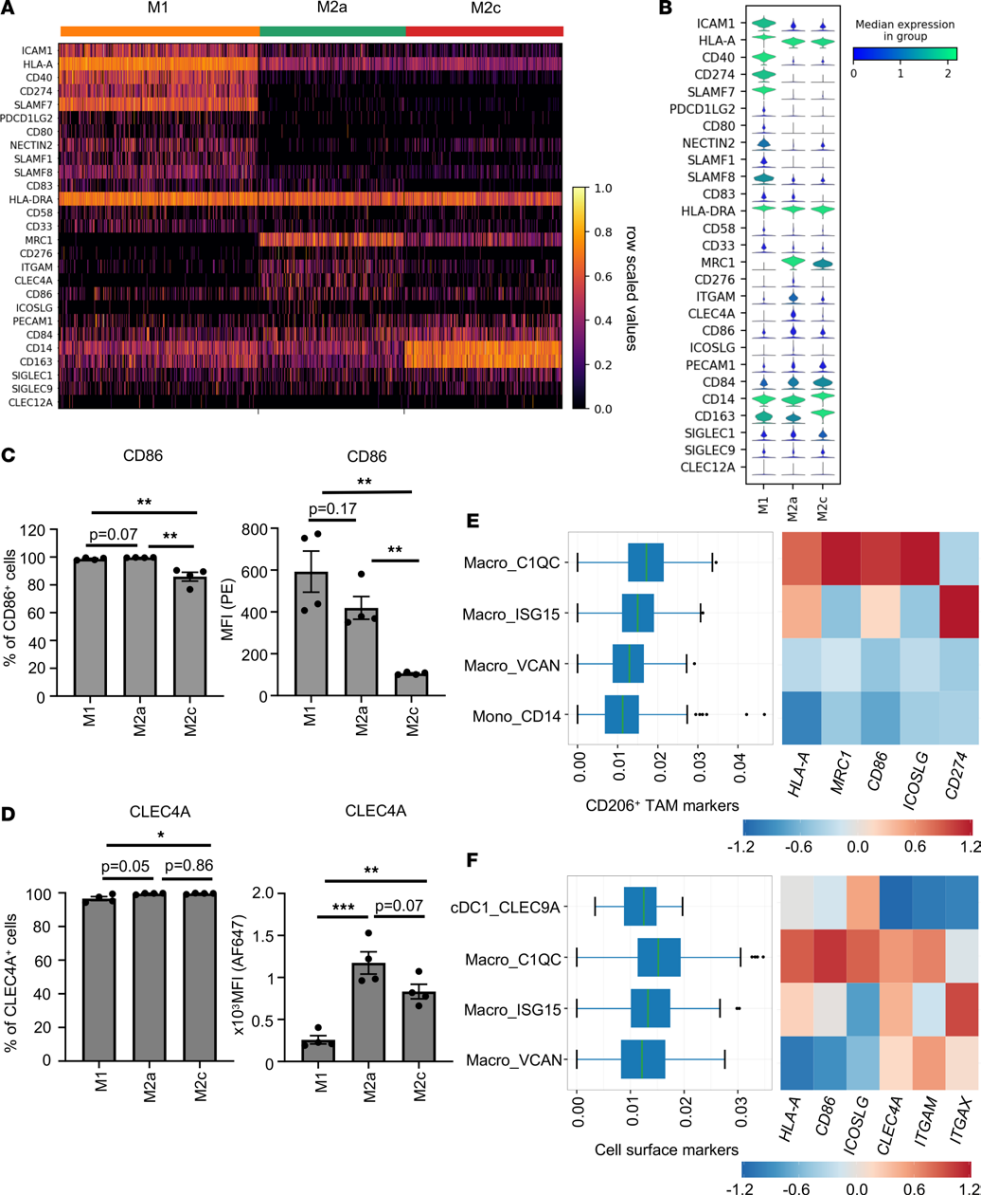
CD206^+^ macrophages express a unique cell surface repertoire. (**A**) Heatmap showing scaled gene expression for selected genes derived from scRNA-Seq macrophage clusters M1, M2a, and M2c. Columns represent individual cells. Color scheme is based on row scaled values from minimum expression value as 0 (black) to maximum expression as 1 (yellow). (**B**) Corresponding violin plots illustrate expression distribution of selected genes on the *y* axis across indicated MDM subsets on the *x* axis. The violin plot color represents the median log normalized expression value from minimum value as 0 (blue) to maximum expression value as 2 (green). (**C** and **D**) Bar diagrams show percentage of CD86^+^ cells (**C**) and CLEC4A^+^ cells (**D**) with corresponding MFI as analyzed by flow cytometry. Data show mean ± SEM (*n* = 4 donors). Unpaired Student’s *t* test, followed by Holm-Šídák test for multiple comparisons, was performed. *P < 0.05, **P < 0.01, ***P < 0.001. (**E** and **F**) The box plot indicates the expression levels of indicated marker genes per cell, displaying the variation of cells in each group (left). Heatmap of *Z* scored median expression of indicated genes per group (right). Color scheme is based on *Z* scored median expression. Minimum expression value as –1.2 (blue) to maximum expression as +1.2 (red). The analysis was performed using scDVA tool.

Our scRNA-Seq data show that all MDM subsets expressed *HLA-A* (Figure 7, A and B). We next analyzed the expression of costimulatory receptors on MDM subsets. Our data show that M1 expressed higher levels of costimulatory receptors *CD40*, *CD80*, and *CD83* compared with alternatively activated M2a or M2c (Figure 7, A and B, and Supplemental Figure 9, A and B) similar to previous reports (25–27). However, expression of costimulatory receptors such as CD86 and ICOSLG were higher in CD206^+^ M2a macrophages (Figure 7, A–C, and Supplemental Figure 9, A–C). In addition, M2a macrophages expressed significantly lower levels of inhibitory ligands PD-L1 (CD274) and PD-L2 (*PDCD1LG2*) compared with M1 macrophages (Figure 7, A and B, and Supplemental Figure 9, A, B, and D).

Altogether, our data show that CD206^+^ M2a macrophages elicit high expression of relevant costimulatory receptors CD86 and ICOSLG along with MHC I, and low expression of PD-L1, a surface marker profile consistent with the observed effective stimulation of CD8^+^ T cells following Ag cross-presentation.

Several C-type lectin receptors, including CLEC4A, CLEC9A, and CLEC12A, have recently been studied for their role in cross-presentation (10, 28, 29). Our data show that all MDM subsets expressed CLEC4A, as analyzed by flow cytometry, although CD206^+^ M2a macrophages expressed high levels of CLEC4A, compared with M1 or M2c macrophages (Supplemental Figure 9B and Figure 7, A, B, and D). We have also analyzed the expression of other C-type lectins known to mediate Ag cross-presentation by DCs, including CLEC9A and CLEC12A (28, 29). Expression of CLEC9A and CLEC12A was generally low on MDM subsets, although it was most prominent on M2a macrophages (Supplemental Figure 9, E and F).

We have also analyzed the expression of costimulatory receptors CD86 and ICOSLG on TAM from B16-F10 melanoma and CT26 colon carcinoma models. In both tumor models, CD11b^+^F4/80^hi^ TAM that coexpress high levels of CD206 also showed significantly high expression of costimulatory receptors CD86 and ICOSLG compared with the CD11b^+^F4/80^dim^ TAM population (Supplemental Figure 10). Moreover, expression levels of CD86 as well as ICOSLG on CD11b^+^F4/80^hi^CD206^+^ TAM were significantly higher in delayed-growth tumors compared with rapid-growth tumors (Supplemental Figure 10).

Lastly, we confirmed if CD206^+^ human TAM from melanoma patients express a similar cell surface receptor repertoire as CD206^+^ M2a MDM. Detailed analysis of tumor-infiltrating myeloid cells from melanoma patients revealed that the CD206^+^ Macro_C1QC subset also expressed high levels of *HLA-A* (MHC I) and *CD86*, as well as *ICOSLG,* and low levels of *CD274*, as we had observed in our cross-presenting CD206^+^ M2a macrophages (Figure 7, E and F). Interestingly, expression levels of MHC I (*HLA-A*) and costimulatory receptors such as *CD86*, *ICOSLG*, and *CLEC4A* were high in tumor-associated CD206^+^ Macro_C1QC even compared with tumor-associated cDC1_CLEC9A (Figure 7F).

The high expression of well-known costimulatory receptors such as ICOSLG and CD86 on CD206^+^ cross-presenting macrophages is in line with a significant positive correlation between *MRC1* and *CD8* expression in various human tumors, including skin cutaneous melanoma, colon adenocarcinoma, and breast invasive carcinoma (Supplemental Figure 11, A–C). We have also observed a significant positive correlation between *MRC1* and effector CD8^+^ T cell markers including *CX3CR1*, *FGFBP2*, *FCGR3A*, and *GZMB* (Supplemental Figure 11, A–C).

Thus, in silico analysis of tumor patient data further corroborates the characteristic phenotype of cross-presenting CD206^+^ macrophages and functional outcomes of successful cross-presentation resulting in enhanced CD8^+^ T cell response, and it identifies CD206^+^ TAM as one of the major cross-presenting subsets found in tumor-infiltrating myeloid cells.

## Discussion

An efficient CD8^+^ T cell response is viewed as critical for successful cancer immunotherapy. Tumor Ag–specific activation of CD8^+^ T cells is primarily dependent on the ability of APC to cross-present tumor Ag that leads to subsequent cross-priming of CD8^+^ T cells. As one approach to immunotherapy for many solid tumors, including melanoma, DC vaccines are currently being investigated for their ability to initiate TAA-specific CD8^+^ T cell responses via effective cross-presentation. However, clinical trials involving DC vaccines have shown limited therapeutic efficiency. Thus, clinical data suggest that cross-presentation by DCs may not, in itself, be sufficient for enabling effective antitumor immunity in many patients (30, 31). TAM typically represent the largest fraction of tumor-associated myeloid cells. Macrophages are known to elicit potent phagocytic capacity, which is a required first step in the process of Ag cross-presentation. However, somewhat surprisingly so, human TAM have not rigorously been studied regarding their possible TAA cross-presentation abilities. To the best of our knowledge, prior reports on this subject essentially are limited to 2 relevant studies: one study suggests that lymph node sinusoidal CD169^+^ macrophages in mice could cross-present dead cell–derived tumor Ag to CD8^+^ T cells, although the efficiency of cross-presentation on a cellular level was not investigated; one other study investigating human CD11c^+^ ascites macrophages suggested that these cells have a limited capacity to provide costimulatory signals to CD8^+^ T cells (32, 33).

So far, Ag cross-presentation readouts for in vitro assays have been largely dependent on the ability of APC to activate Ag-specific CD8^+^ T cells using model Ag (32, 34). To directly assess and quantify an ability of APC to cross-present Ag, we have used the TCRL D7 antibody that specifically recognizes melanoma-associated TyrD Ag epitope TyrD_369–377_ (16). Due to their TCR-like specificities, TCRL antibodies can be used for detection of 1 specific peptide/MHC I complex and to therapeutically target tumor cells that express this specific complex (35). We have redevised the use of such TCRL D7 antibody to directly quantify the Ag cross-presenting abilities of macrophages and cDC1. Quantitative analysis of TyrD_369–377_/HLA-A02 complexes suggests that CD206^hi^ macrophages are more efficient than CD206^dim^ or CD206^lo^ macrophages in cross-presentation of TyrD Ag (Figure 1 and Figure 4). Additionally, our data with short TyrD peptide show that M1 macrophages were significantly less capable of stimulating TyrD-specific CD8^+^ T cells compared with M2a and M2c macrophages, even though loading experiments with short TyrD peptide had shown that M1 macrophages could accept a higher peptide load on a per-cell basis (Figure 4, A, B, and E). These data suggest that M1 macrophages may be inferior stimulators of Ag-specific CD8^+^ T cells compared with M2a macrophages. Indeed, our data show that M1 macrophages expressed significantly higher levels of inhibitory ligand PD-L1 and PD-L2 compared with M2a macrophages (Figure 7, A and B, and Supplemental Figure 9, A, B, and D). Furthermore, Ag-primed CD8^+^T cells may acquire an anergic unresponsive phenotype when they are exposed to high numbers of peptide/MHC complexes (36). Thus, the reduced T cell–stimulating ability of M1 macrophages may result from high expression of inhibitory ligands like PD-L1 and PD-L2 and/or high abundance of peptide/MHC I complexes on their cell surface (36, 37). Furthermore, using TCRL D7 antibody, we have provided direct evidence that CD206^+^ MDM are capable of cross-presenting TyrD Ag at an efficiency at least comparable with cDC1 on the individual cell level; after cross-presentation of long TyrD peptide, approximately 13% of CD206^+^ M2a MDM were recognized by the TCRL D7 antibody, roughly 5-fold over control, whereas approximately 6% of cDC1 were recognized by the TCRL D7 antibody, roughly 3-fold over control (Figure 4D and Supplemental Figure 7B). Furthermore, approximately 13% of TyrD_369–377_/HLA-A02^+^ CD206^+^ macrophages induced approximately 500 pg/mL IFN-γ per 25,000 total CD8^+^ T cells, whereas approximately 6% TyrD_369–377_/HLA-A02^+^ cDC1 cells induced 800 pg/mL IFN-γ per 25,000 total CD8^+^ T cells (Figure 4F and Supplemental Figure 7D). These data suggest superior Ag cross-presentation ability of CD206^+^ macrophages, whereas cDC1 appear more effective in Ag-specific CD8^+^ T cell stimulation (Figure 4 and Supplemental Figure 7). Thus, the 2 readouts of TCRL D7 antibody and TyrD-specific CD8^+^ T cells allowed us to assess the ability of APC including TAM, MDM, and cDc1 to cross-present Ag and to stimulate Ag-specific CD8^+^ T cell responses in independent but complementary assays. In a similar set of experiments, we demonstrated that in vitro–generated CD206^+^ human TAM are capable of TyrD cross-presentation, both at the level of cell surface detection of TyrD_369–377_/HLA-A02 and at the level of TyrD-specific CD8^+^ T cell activation. Analysis of preexisting data sets suggested specific expression of CD206 by a TAM subset characterized by expression of C1QC in melanoma patients (Figure 1). Previous studies have reported an inflammatory phenotype of M2-like C1QC^+^ macrophages in colorectal carcinoma (38). In silico analysis of correlative cell-to-cell interaction networks and ligand-receptor interactions have suggested a role of C1QC^+^ TAM in recruiting as well activating CD8^+^ T cells (38). Furthermore, analysis of tumor-infiltrating myeloid cells showed that CD206^+^C1QC^+^ TAM express high levels of MHC I and costimulatory receptors such as *CD86* and *ICOSLG* compared with tumor-associated cDC1 (Figure 7). Moreover, the high expression of *MRC1* and high proportion of M2 macrophages but not of activated DCs significantly correlated with better overall survival in melanoma patients (Figure 1). Based on these data, it is tempting to hypothesize that CD206^+^C1QC^+^ TAM represent a dominant cross-presenting CD206^+^ TAM population in melanoma patients. Data from the B16-F10 and CT26 syngeneic tumor models have shown that mice with delayed-growth tumors elicited significantly higher numbers of cross-presenting TAM, phenotypically characterized by CD11b^+^F4/80^hi^CD206^+^, compared with mice with rapid-growth tumors (Figure 2 and Supplemental Figure 3). CD206^+^ TAM from delayed-growth tumors were more efficient in stimulating Ag-specific CD8a^+^ T cells compared with TAM from rapid-growth tumors (Figure 2, Figure 3, and Supplemental Figure 5). Further corroborating the Ag cross-presenting ability of CD206^+^ TAM, we have observed a significant positive correlation between *MRC1* expression and expression of effector cytotoxic CD8^+^ T cells markers such as *CX3CR1* and Granzyme B (*GZMB*) in various cancers including cutaneous melanoma, breast carcinoma and colon adenocarcinoma (Supplemental Figure 11). Together, these data suggest that CD206^+^ TAM subsets should be noted for their ability to effectively cross-present Ag and efficiently stimulate Ag-specific CD8^+^ T cells. Furthermore, comparative analysis between in vitro–generated CD163^lo^CD206^hi^ M2a macrophages and CD163^hi^CD206^lo^ M2c macrophages have clearly defined efficient Ag cross-presentation by CD206^hi^ M2a macrophages, similar to CD206^hi^ TAM (Figure 1, FIgure 2, Figure 3, Figure 4). Thus, high surface expression of CD206 by macrophages may be viewed as an overarching phenotypic marker of cross-presenting macrophage subsets. Indeed, a subset of mouse Kupffer cells capable of cross-priming Hepatitis B Ag-specific CD8^+^ T cells in response to exogenous IL-2 was found to be CD206^+^ (39), extending the role of CD206 as a phenotypic marker for identifying the tissue-resident macrophage subsets with a cross-presenting ability in different biological context of chronic viral infections. Previous studies investigating the origin of TAM have confirmed that the majority of TAM differ from tissue-resident macrophages by transcriptional profile and, thus, most probably arise from circulating monocytes (38, 40, 41). Therefore, the majority of TAM, including CD206^hi^ cross-presenting TAM, is likely to be derived from circulating monocytes, although genetic lineage tracing studies — e.g., using MS4A3 as a marker — would have to be performed to assess this at the highest possible level of resolution (42).

To further validate our findings, we decided to study Ag cross-presentation with an unrelated Ag, CMV pp65. CMV pp65 protein originates from cytomegalovirus; therefore, it served not only as an unrelated Ag, but also to interrogate a different biological context of cross-presentation — in this case, mimicking the adaptive immune response against an infectious agent (i.e., non–self-Ag). Using soluble CMV pp65 Ag, we have confirmed the ability of CD206^+^ M2a macrophages to cross-present Ag, as evident by significantly superior cytotoxicity of CD206^+^ M2a-stimulated CMV-specific CD8^+^ T cells (Figure 6). These data differ from a recent study suggesting the inability of macrophages, including ascites macrophages, to induce Ag-specific effector CD8^+^ T cells (32). Of note, only CD11c^+^ macrophages had been analyzed in this study, and CD206^+^ M2a as well as Macro_C1QC TAM were found to be CD11b^+^CD11c^–^ (Figure 7) (32, 43).

Tumor cells undergo cell death at variable but generally low rates. In addition, irradiation and use of anticancer drugs as a part of standard-of-care treatment regimens lead to tumor cell death. Dead tumor cells can also serve as a source of TAA. Therefore, we interrogated the ability of macrophages to cross-present cell-derived TAA. In our in vitro experimental set up, we could not observe cross-presentation of cell-derived Ag and subsequent stimulation of Ag-specific CD8^+^ T cells by MDM subsets (Figure 4). The lack of induction of an Ag-specific CD8^+^ T cell response did not result from inefficient uptake of Ag in cell-derived form (Figure 5). Of note, MDM express very low levels of *RAB43*, a key molecule for cross-presentation of cell-derived Ag by CD8α^+^ DCs (44). Thus, MDM, including CD206^+^ macrophages, may not be perfectly equipped to cross-present cell-derived Ag. Finally, we cannot rule out a scenario in which the level of cross-presented TyrD was too low to be clearly detectable by our readouts, simply due to the possibility that TyrD Ag was in competition with other HLA-A02–compatible and cell-associated Ag present in large abundance. The lack of detectable Ag-specific CD8^+^ T cell activation might also result from apoptosis-associated and tolerance-inducing cell surface receptor expression by dying tumor cells (45).

Nonetheless, our data with 2 unrelated soluble Ags suggest that CD206^+^ M2a macrophages are efficient in cross-presentation as well as activation of Ag-specific CD8^+^ T cells. At first glance, these results appear at odds with the reported pro- and antiinflammatory role of M1 and M2a macrophages, respectively. However, it is worth mentioning that, so far, M1 and M2a macrophages have been characterized predominantly based on their cytokine and surface marker profile, and the ability of macrophage subsets to stimulate T cells has not been systematically studied before (25, 27). Of note, there is no plausible reason why the actual process of Ag cross-presentation should necessarily be viewed as proinflammatory. However, the consequence of successful cross-presentation could result in Ag-dependent controlled inflammation in the context of CD8^+^ T cell effector function. Moreover, many previous studies investigating the role M2 macrophages in the tumor microenvironment have characterized those macrophages based on expression of CD68 and CD163. The detailed reanalysis of TAM population based on CD206 expression have identified the CD206^+^ TAM population to be associated with pronounced lymphocyte infiltrate and improved survival in breast cancer patients (5, 15).

Endocytosis receptors such as C-type lectin receptors, including mannose receptor (CD206), have been previously shown to target internalized soluble Ag to endosomal compartments, thereby providing favorable conditions for Ag cross-presentation (10, 28, 29, 46). Our data indeed show that M2a, as well TAM, express high levels of CD206 and C-type lectin receptors, including CLEC4A (Figure 7 and Supplemental Figures 2, 6, and 9). In fact, melanoma-associated CD206^+^ Macro_C1QC cells expressed higher levels of MHC I, more costimulatory receptors, and more C-type lectin CLEC4A receptor than melanoma-associated cDC1_CLEC9A cells (Figure 7). Expression of these endocytosis- and cross-presentation–associated receptors by macrophages provided indirect phenotypic evidence that CD206^+^ macrophage subsets should be included in the discussion of APC with cross-presentation abilities. Furthermore, therapeutic targeting of these receptors on macrophage subsets may aid future immune-oncology or vaccine approaches, success of which may be linked to effective cross-presentation (47).

In summary, we provide the first systematic study to our knowledge to impart CD206^+^ MDM subsets, including TAM, for their previously unrecognized capabilities of Ag cross-presentation and efficient Ag-specific CD8^+^ T cell activation. Peptide-based vaccines are being extensively studied for their therapeutic use in infectious diseases, as well as in cancers, aiming to maximize Ag-specific T cell responses (48, 49). Success of such peptide-based vaccination is largely dependent on the Ag cross-presentation ability of APC from patients (50). Our study expands the portfolio of immune-modulatory properties of macrophages beyond the well-described activities in phagocytosis and the production of antimicrobial mediators and cytokines. Based on these insights, future strategies for cancer immunotherapy and vaccine development should consider macrophages’ cross-presentation ability and assess macrophages as candidate cellular targets. In addition, macrophage-dependent cross-presentation of self-Ags in the context of tissue inflammation may be considered as a possible factor in the pathogenesis of autoimmune conditions with prominent CD8^+^ T cell contributions (51, 52).

## Methods

Supplemental Methods are available online with this article.

### Animals.

Male C57BL/6 mice, 6–8 weeks old, were obtained from Taconic Biosciences. BALB/cAnNCrl, 6–8 weeks old, were purchased from Charles River Laboratories. Mice were maintained under controlled environmental conditions including temperature (22°C ± 2°C), light (12-hour light/dark cycle), and relative air humidity (55% ± 10%). Animals were housed in group with food and water ad libitum. After 7 days of adaptation at the Charles River vivarium, either B16-F10/B16-F10–OVA melanoma cells or CT26 colon carcinoma cells (0.3 × 10^6^ cells /100 μL/mouse) were s.c. injected into the right flank of animals. The tumor volumes were determined 3 times per week by 2-dimensional measurement with a digital caliper (S_Cal EVO Bluetooth). Tumor volumes were calculated according to the formula: tumor volume = (l × w²) × π/6, where l = largest diameter and w = width (perpendicular diameter) of the tumor (in mm). TCR (TCRα-V2 and TCRβ-V5) transgenic C57BL/6 (OT-I) mice were obtained from Charles River Laboratories.

### Isolation of TAM.

The tumors were resected at indicated time points, and extracted tumors were maintained in ice-cold PBS supplemented with 0.5% BSA until further use. The single-cell suspension was generated using Tumor Dissociation Kit, mouse (Miltenyi Biotec) as per the manufacturer’s protocol. Live cells from single cell suspension were enriched using Dead Cell Removal Kit (Miltenyi Biotec) according to the manufacturer’s protocol. The cells were then stained with Fixable Viability Stain (FVS780) at room temperature. The cells were labeled at 4°C with respective antibodies. TAM were isolated using BD FACSAria III cell sorter (BD Biosciences).

### Generation of MDM.

Fresh blood was obtained from healthy volunteers. PBMC were isolated from heparinized fresh blood by standard density gradient centrifugation (300*g*, 20 minutes; temperature 21°C) with Ficoll-Paque Plus (GE Healthcare). CD14^+^ monocytes were obtained by negative selection using Human Monocyte Isolation Kit (Stemcell Technologies). Purity of monocyte population was checked regularly and was found to be ≥ 96%. CD14^+^ monocytes were cultured at 1 × 10^6^ cells/mL in complete RPMI 1640 medium in UpCell plates (Thermo Fisher Scientific). To trigger MDM generation, cells were cultured with 100 ng/mL M-CSF (Peprotech) for 5 days. At day 5, cells were stimulated for 48 hours with 50 ng/mL LPS-EB Ultrapure (Invivogen) and 20 ng/mL IFN-γ (Peprotech) for M1 differentiation, with 20 ng/mL IL-4 and IL-13 (R&D Systems) for M2a differentiation, and with 20 ng/mL IL-10 (BioLegend) for M2c differentiation. At day 7, the adherent MDM (M1, M2a, and M2c) were collected. The viability of differentiated cells was routinely checked using Trypan Blue (Invitrogen) and was found to be ≥ 92%.

### In vitro generation of TAM.

TAM were generated in vitro from CD14^+^ monocytes as described previously (18). Briefly, isolated monocytes were cultured in complete RPMI 1640 medium and tumor cell (MDA-MB231, ATCC) conditioned medium in 1:1 ratio. The culture medium was supplemented with cytokine cocktail containing M-CSF (100 ng/mL), IL-4 (100 ng/mL), and IL-10 (100 ng/mL). The cells were supplemented with fresh culture medium containing cytokine cocktail after 3 days. The cells were cultured for 7 days. The viability of differentiated cells was routinely checked and was found to be to be ≥ 85%.

### Antigenic protein and peptides.

TyrD short (9 amino acids) peptide (YMDGTMSQV), Scr short peptide (MQMSYTVDG), TyrD long (27 amino acids) peptide (ASQSSMHNALHIYMDGTMSQVQGSAND), and Scr long peptide (AQVQGNDSQMDMSIYMHGTSASSNALH) were synthesized and purchased from JPT Peptide technologies. The purity of synthesized peptides was checked routinely using HPLC and was found to be ≥ 96%. The 27–amino acid peptides were used as a source of soluble TAA. CMV pp65 peptide (NLVPMVATV) was purchased from IBA Lifesciences. CMV pp65 recombinant protein was purchased from Miltenyi Biotec. OVA Ag peptide (SIINFEKL), OVA, and BSA protein were purchased from Sigma Aldrich. For pHrodo dye labeling, TyrD long peptide with additional lysine residues were used (KASQSSMHNALHIYMDGTMSQVQGSANDK). TyrD peptide was labeled using pHrodo Red Microscale Labeling Kit (Thermo Fisher Scientific) as per manufacturer’s protocol.

### OVA Ag cross-presentation assay.

The sorted TAM (20 × 10^3^ cells/well) were incubated with 1 mg/mL soluble OVA or BSA protein for 16 hours or with 3 ng/mL OVA Ag peptide for 1 hour at 37°C. TAM were cultured in IMDM supplemented with Glutamax, MEM nonessential amino acids, and sodium pyruvate. The cells were then washed twice using culture medium. Naive CD8a^+^ T cells were isolated from splenocytes of C57BL/6 OT-I transgenic mice using Naive CD8a^+^T Cell Isolation Kit, mouse (Miltenyi Biotec), according to the manufacturer’s protocol. The purity of isolated cells was checked routinely and was found to be ≥ 95%. The cells were then labeled with Cell Trace Violet (CTV) (Thermo Fisher Scientific) according to the manufacture’s protocol. The CTV labeled naive CD8a^+^ T cells (1 × 10^5^ cells/well) were then added to TAM. The cells were analyzed for proliferation after approximately 80 hours of culture using LSRFortessa X20 (BD Biosciences). Analysis was performed using Peak modelling software FlowJo, and the percentage of proliferating CD8a^+^ T cells was determined.

### Culturing of TyrD-specific T58-CD8^+^ T cells.

Human CD8^+^ T cells expressing high-avidity TyrD_369–377_–specific T cell receptor (T58) have been reported previously (17). T58 cells were provided by E. Noessner (Helmholtz Zentrum München, Germany). T58 cells were cultured in RPMI 1640 supplemented with 1× glutamax, 1× sodium pyruvate, 1× MEM nonessential amino acids (Thermo Fisher Scientific), 10% human serum (Sigma Aldrich), and 50 U/mL IL-2 (Peprotech).

### Preparation of COLO829 cells for efferocytosis assay.

COLO829 cells were seeded in a 6-well plate (Thermo Fisher Scientific) at a density of 1 × 10^6^ cell/well and were rested for 3 hours. Cells were then treated with 302 nm UV for 10 minutes. After UV treatment, cells were incubated overnight and then harvested. A fraction of cells was used to analyze the induction of cell death by Annexin V/7-AAD staining (BD Biosciences). The rest of the cells were labeled with pHrodo dye using Incucyte pHrodo Red Cell Labeling Kit (Essen Biosciences) according to manufacturer’s protocol.

### scRNA-Seq for MDM subsets.

MDM were generated from PBMC-derived monocytes from 2 healthy volunteers. M1, M2a, and M2c macrophages were collected 24 hours later upon addition of respective differentiation cytokines as described before. Cells were evaluated for viability (>90%), aggregate (<5%), and cell concentration using the Nucleocounter NC-200 (Chemometec). Single-cell transcriptomics libraries were generated via the Chromium Controller and the Single Cell 3′ Reagent Kit v3 (10X Genomics) according to the manufacturer’s instructions. Briefly, approximately 8700 cells per sample were loaded onto a B Chip, aiming to capture 5000 cells, with an expected 4.6% doublet rate. The Chromium Controller ran for droplet generation, and reverse transcription was performed in the droplets. In total, 80 ng of cDNA was used for the gene expression library preparation. Samples were prepared according to the 10X Genomics’ protocol, except for an additional 1× SPRISelect Beads (Beckman Coulter) to ensure full removal of primer and adaptor dimers prior to the final elution for gene expression libraries. Libraries were quantitatively and qualitatively assessed using the Qubit 4 Fluorometer (Thermo Fisher Scientific) and the Fragment Analyzer (Agilent), respectively. Gene expression libraries were 440 bp in average length. Libraries were normalized, pooled, and sequenced on an Illumina HiSeq 4000. Single-cell data were processed using CellRanger v3.0.2. Reads were aligned to human reference transcriptome GRCh38.93 from Ensembl. Count matrices were processed for each sample individually. Cells were filtered using Scanpy v.1.5 with the following parameters: minimum number of genes, 500; maximum number of genes, 3500; maximum fraction of mitochondrial genes, 0.20. On average, each sample contained 3500 cells after filtering for a total of 21,000 cells. Standard processing of single-cell data was carried out following the best practices outlined previously (53). All 6 samples were concatenated and total UMI counts per cell were normalized using SCRAN and then converted to log (normalize values + 1). Subsequently, highly variable genes (HVG) were identified, and principal component analysis (PCA) was computed using the Seurat 2 method. UMAP was computed using 50 PCAs. In the resulting UMAPs, we could validate that all 2 replicates clustered together for the 3 conditions. scRNA-Seq data for all samples included in this study can be accessed in NCBI’s Gene Expression Omnibus (GEO; GSE199378).

### In silico analysis of gene expression in human melanoma tumor samples.

The pan-cancer scRNA-Seq data visualization and analysis (scDVA) platform (panmyeloid.cancer-pku.cn) was used to perform analysis of published scRNA-Seq data for myeloid cells including TAM subsets in melanoma (54). The database Gene Expression Profiling Interactive Analysis (GEPIA, http://gepia.cancer-pku.cn/index.html) was used to generate survival plots for overall survival based on gene expression and cell proportion in melanoma and in renal cell carcinoma patients using TCGA data sets (55, 56). Gene expression correlation analysis was performed on TCGA expression data using GEPIA. The Spearman method was used to determine the correlation coefficient.

### Flow cytometry analysis.

For cell surface staining, cells (2 × 10^5^) were incubated with conjugated mAbs for 30 minutes at 4°C. Nonspecific binding was blocked by using 1% Fc block (Miltenyi Biotec). Cells were stained with indicated antibodies or respective isotype control antibodies where indicated. See Supplemental Tables 1 and 2 for the list of antibodies used for flow cytometry.

For intracellular staining Intracellular Fixation and Permeabilization Buffer Set (eBioscience) was used. Briefly, the cells were fixed in fixation buffer at room temperature for 40 minutes. The cells were then stained with indicated antibodies in permeabilization buffer at room temperature for 30 minutes. The cells were washed extensively.

For unconjugated TCRL D7 antibody, PE-conjugated rat anti–mouse IgG2a antibody (Invitrogen) was used as the second-step reagent. For detection of TyrD_369–377_/MHC I peptide complex using TCRL D7 antibody, MDM from HLA-A02^+^ donors were used. To analyze Ag loading, MDM, TAM, or T2 cells (2 × 10^5^ cell/condition) were treated with short TyrD or Scr peptide (20 μM) for 1 hour at 37°C. To analyze Ag cross-presentation, cells were treated with long TyrD or Scr peptide (20 μM) for 16 hours. The cells were washed extensively. Cells were then stained with TCRL D7 antibody and second step PE-conjugated rat anti–mouse IgG2a antibody as described above (Supplemental Tables 1 and 2).

For tetramer stainings (Supplemental Table 3), cells were stained with indicated mAbs and with either respective tetramer or control tetramer at 4°C as described above. Flow cytometry analyses were performed using either LSRFortessa X20 (BD Biosciences) or Attune NxT Acoustic Focusing Cytometer (Thermo Fisher Scientific).

### Ag-specific stimulation of T58 CD8^+^ T cells.

MDM, cDC1, or TAM (5 × 10^3^ cells/well) from HLA-A02^+^ donors were coincubated with short peptide (1 hour), long peptide (16 hour), or UV-treated COLO829 cells (15 × 10^3^ cells/well; 5 hours) in a round-bottom 96-well plates (Corning). The cells were washed extensively. T58 cells were then added to Ag-stimulated myeloid cells at 5:1 ratio (T58 cells/myeloid cells) and were cultured for 4 days. At day 4, supernatants were harvested and were pooled from triplicate wells. Supernatants were collected and kept at –80°C until measurement of IFN-γ concentration by CBA (BD Biosciences).

### Endocytosis assay.

Differentiated MDM were harvested, washed, and were seeded in 96-well Clear Flat Bottom plates with low evaporation lid (Corning) (25 × 10^3^ cells/well). The cells were rested and were allowed to settle for 1 hour, as described previously (23). pHrodo Red–labeled TyrD long peptide and pHrodo Red–labeled Dextran (Thermo Fisher Scientific) were added to respective wells. pHrodo Red–labeled UV-treated COLO829 cells were added to MDM at 3:1 ratio (75 × 10^3^ COLO829/well; 25 × 10^3^ MDM/well). Confluence of seeded cells was analyzed at 0 hours using Incucyte S3 live cell analysis system (Essen Biosciences). For calculating confluence, wells with untreated MDM were analyzed. Uptake by MDM subsets was analyzed over time (0–18 hours) using Incucyte S3 Live-Cell Analysis System.

### Activation and expansion of CMV-specific CD8^+^ T cells.

CMV-specific activation and expansion was performed as described previously (24). Briefly, CD8^+^ T cells were isolated from PBMC by negative selection using Human CD8^+^ T cell isolation kit (Miltenyi Biotec). Isolated CD8^+^ T cells were cultured overnight in complete RPMI 1640 medium containing Glutamax (Thermo Fisher Scientific) supplemented with 10% premium low-endotoxin FBS (Thermo Fisher Scientific) and 5 ng/mL IL-7 (Peprotech). MDM subsets were left untreated or were pretreated with CMV pp65 peptide (for 1 hour) or with CMV pp65 protein (for 16 hours). The T cells were then cocultured with unstimulated or CMV-stimulated MDM subsets in 4:1 (T cells/MDM) ratio in the presence of 30 ng/mL IL-21 (Peprotech) for 72 hours. The cells were then supplemented with new T cell culture medium containing 5 ng/mL IL-15 and IL-7 (Peprotech). The cells were further incubated for 72 hours. Afterwards, T cells were transferred to a new plate and were supplemented with fresh T cell medium containing 5 ng/mL IL-7 and IL-15. The cells were further cultured for 48 hours. The cells were again supplemented with fresh medium containing 10 ng/mL IL-7 and IL-15, and they were further incubated for 72 hours. At the end of day 11, the cells were used for MHC I tetramer staining, as well as for cytotoxicity assay.

### Ag-specific cytotoxicity assay.

For killing assays, colon carcinoma HCT116 cells were used as target cells. HCT116 cells were either kept untreated or were pulsed with CMV pp65 peptide. Unpulsed or pulsed HCT116 cells were then cocultured with Ag-specific CD8^+^ T cells in 3:1 (HCT116/CD8^+^ T cells) ratio. After 2 days of coculture, the viability of cells was assessed using CellTiter-Glo reagents according to manufacturer’s protocol. Killing of target cells was calculated using GraphPad Prism as a percentage of target cell survival normalized to values obtained from HCT116 cells alone.

### Statistics.

Statistical analysis was performed using GraphPad Prism software. Unpaired, 2-tailed Student’s *t* test followed by Holm-Šídák test for multiple comparisons was performed, and *P* < 0.05 was considered significant. Significant values are represented as *P < 0.05, **P < 0.01, and ***P < 0.001. For overall survival analysis, log-rank test with 95% CI was used.

### Study approval.

All studies on human donor blood were performed in accordance with the guidelines and regulations of German legislation, and the experimental protocol was approved by the ethical committee of the Landesärztekammer Baden-Württemberg (Germany). Anonymized blood samples were obtained from healthy volunteers who provided written informed consent.

All animal experiments and procedures were approved by the regulatory authorities for animal welfare (17-005-G, 16-006-O, 21-005-O) and were compiled in accordance with the federal and state policies and Boehringer Ingelheim policies on animal research, which are fully accredited by the Association for Assessment and Accreditation of Laboratory Animal Care (AAALAC).

## Author contributions

MM conceptualized the study, designed and performed the experiments, analyzed the data, and wrote the manuscript. AKM performed CMV cross-presentation experiments. DR performed FACS of TAM. WSW performed RNA isolation and qPCR and reviewed the manuscript. RL performed animal experiments. NS and RK generated and validated TCRL D7 antibody. KL contributed to Ag uptake assays using Incucyte. TM, DK, CV, and FR performed and analyzed scRNA-Seq data. EN provided T58 CD8^+^ T cells and critically reviewed the study and manuscript. JP supervised the tumor model studies and critically reviewed the study and manuscript. KCEK critically reviewed the study and manuscript. LKS and KK provided key reagents and critically reviewed the study and manuscript. SP conceptualized and directed the study and wrote the manuscript.

## Supplementary Material

Supplemental data

## Version 1 05/03/2022

In-Press Preview

## Version 2 06/08/2022

Electronic publication
