# Supplementary material for: CD206+ tumor-associated macrophages cross-present tumor antigen and drive antitumor immunity
Source: JCI Insight. 2022 Jun 8;7(11):e155022. doi: 10.1172/jci.insight.155022 (PMC9220841; doi:10.1172/jci.insight.155022)
Supplement: Supplemental data [file jciinsight-7-155022-s265.pdf]

Supplemental figures and figure legends

Supplemental Figure S1

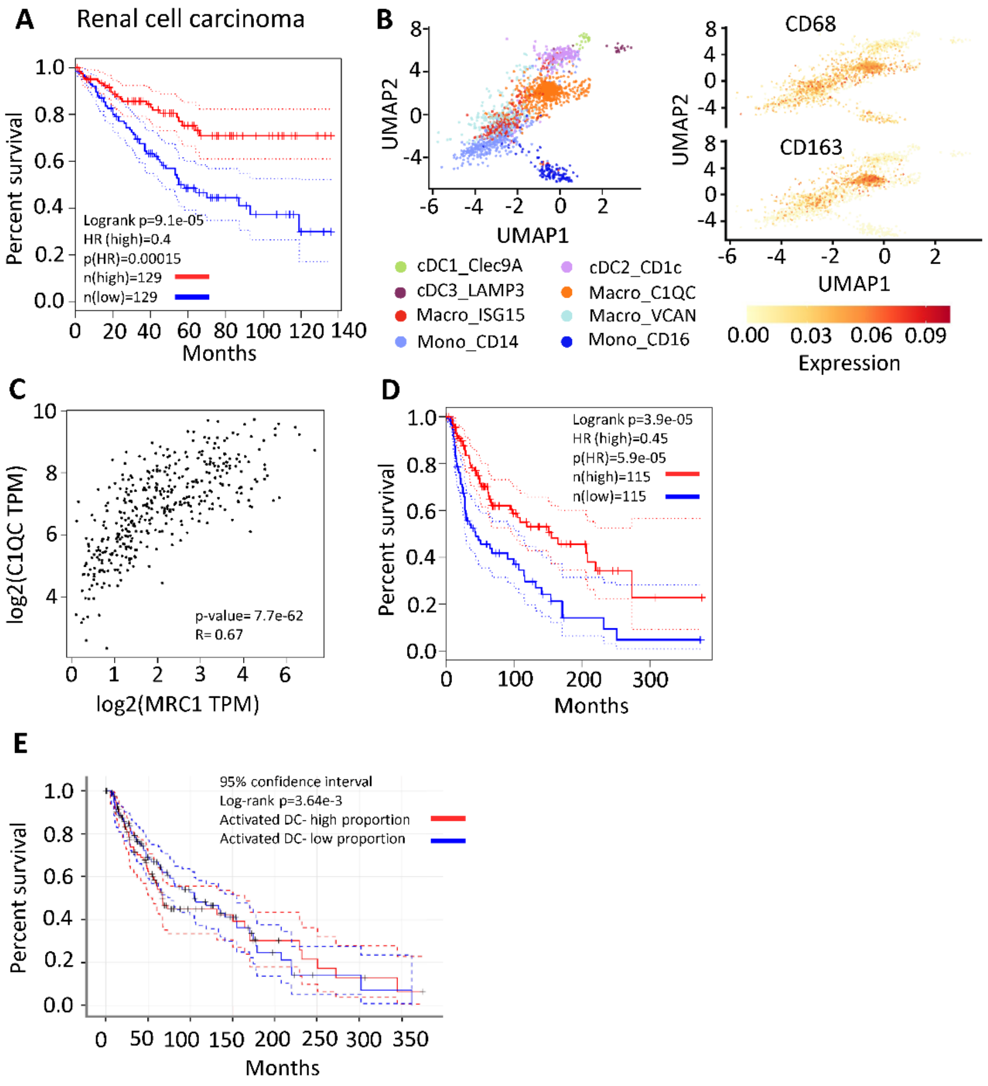

Supplemental Figure S1 In silico characterization of human CD206^{+} tumor-associated macrophages

(**A**) Overall survival analysis based on *MRC1* mRNA expression (blue: low *MRC1* TPM; red: high *MRC1* TPM) in renal cell carcinoma patients (n=258) from TCGA database. (**B**) UMAP plots showing myeloid cell populations in melanoma colored by clusters (left) and expression of *CD68* and *CD163* for cell type annotation (right). The analysis was performed using Pan-cancer single cell RNA-Seq data visualization and

analysis (scDVA) tool. (**C**) Correlation analysis between expression of *MRC1* mRNA and *C1QC* mRNA in melanoma patients (n=469) from TCGA database. (**D**) Overall survival analysis based on *C1QC* mRNA (blue: low *C1QC* TPM; red: high *C1QC* TPM) expression in melanoma patients (n=230) from the TCGA database. (**E**) Overall survival analysis based on proportion of activated dendritic cells in tumor tissue (blue: low dendritic cell proportion; red: high dendritic cell proportion) in cutaneous melanoma patients (n=469) from TCGA database. (**A, C-E**) Analysis was performed using GEPIA.

Supplemental Figure S2

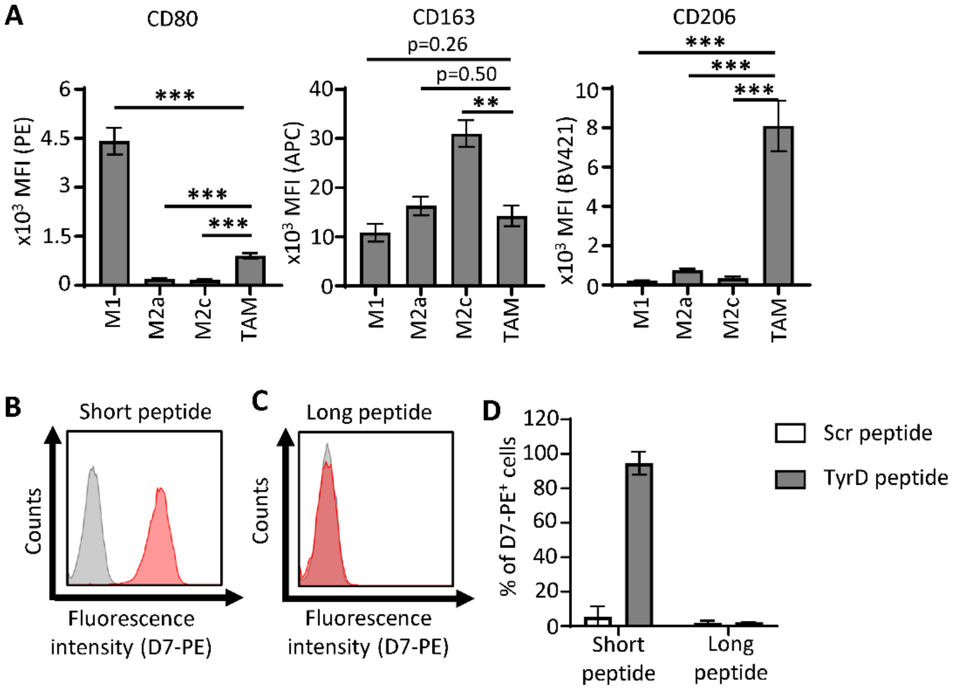

Supplemental Figure S2 Characterization of in vitro generated CD206^{+} TAM and TCR like D7 antibody

(**A**) Bar diagrams represent mean fluorescence intensity (MFI) of cell surface markers CD80, CD163 and CD206 on in vitro generated TAM as analyzed by flow cytometry. Data show mean ± SEM (n= 4 donors). $^{**}P < 0.01$, $^{***}P < 0.001$. (**B**) Detection of HLA-A02 restricted TyrD short peptide (red histogram) or Scr short peptide (grey histogram) with D7 TCRL antibody on HLA-A02^{+} TAP^{-} T2 cells. (**C**) As in (**B**), but T2 cells were treated with TyrD long peptide (red histogram) or Scr long peptide (grey histograms). (**B** and **C**) Data are representative of 2 independent experiments. (**D**) Bar diagram represents percentage of positive cells as detected by reactivity profile of D7 Ab upon treatment of T2 cells with either short peptide (Scr; open bars and TyrD; filled bars) or long peptide (Scr; open bars and TyrD; filled bars). Data show mean ± SEM of two independent experiments.

Supplemental Figure S3

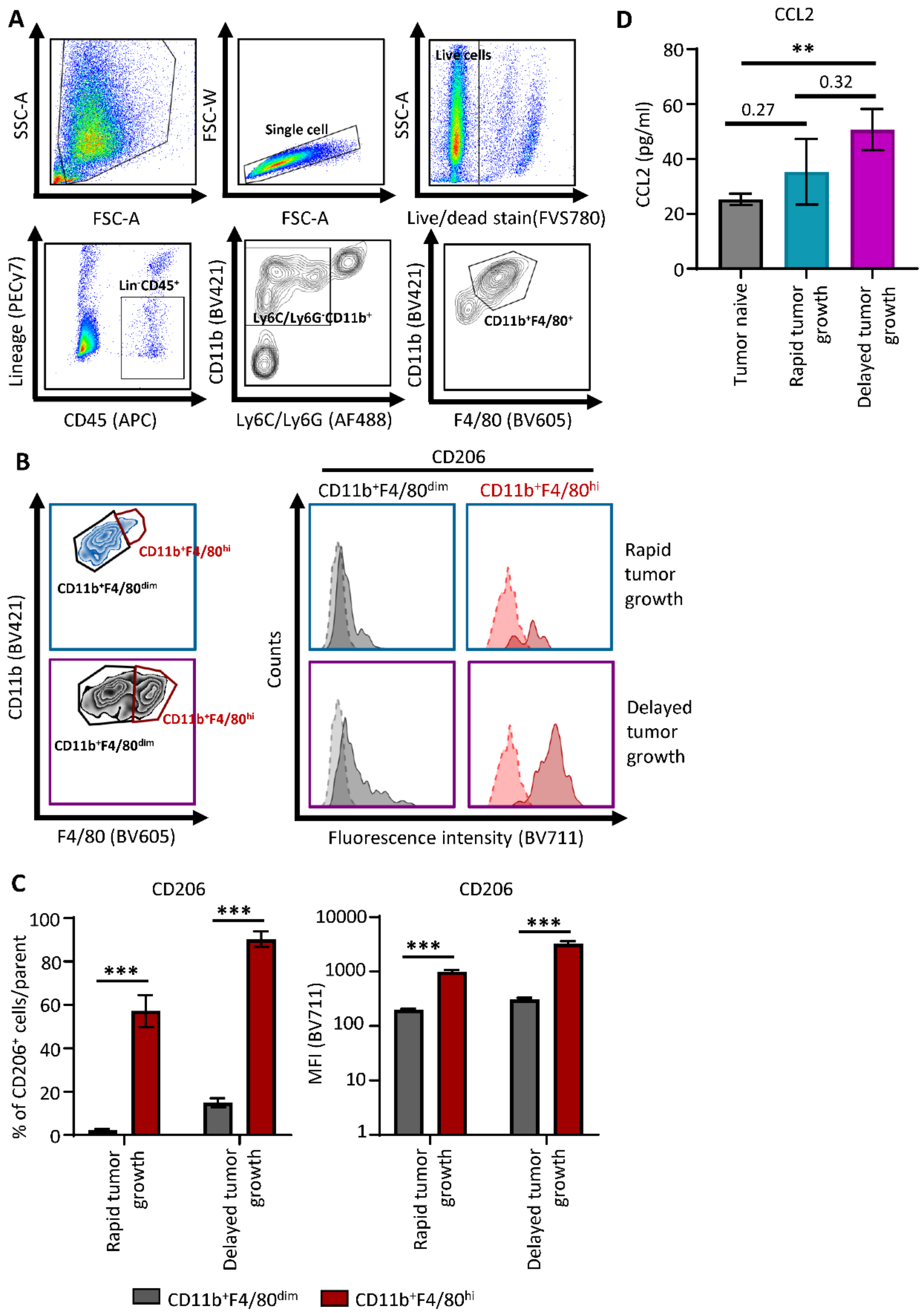

Supplemental Figure S3 (continued)

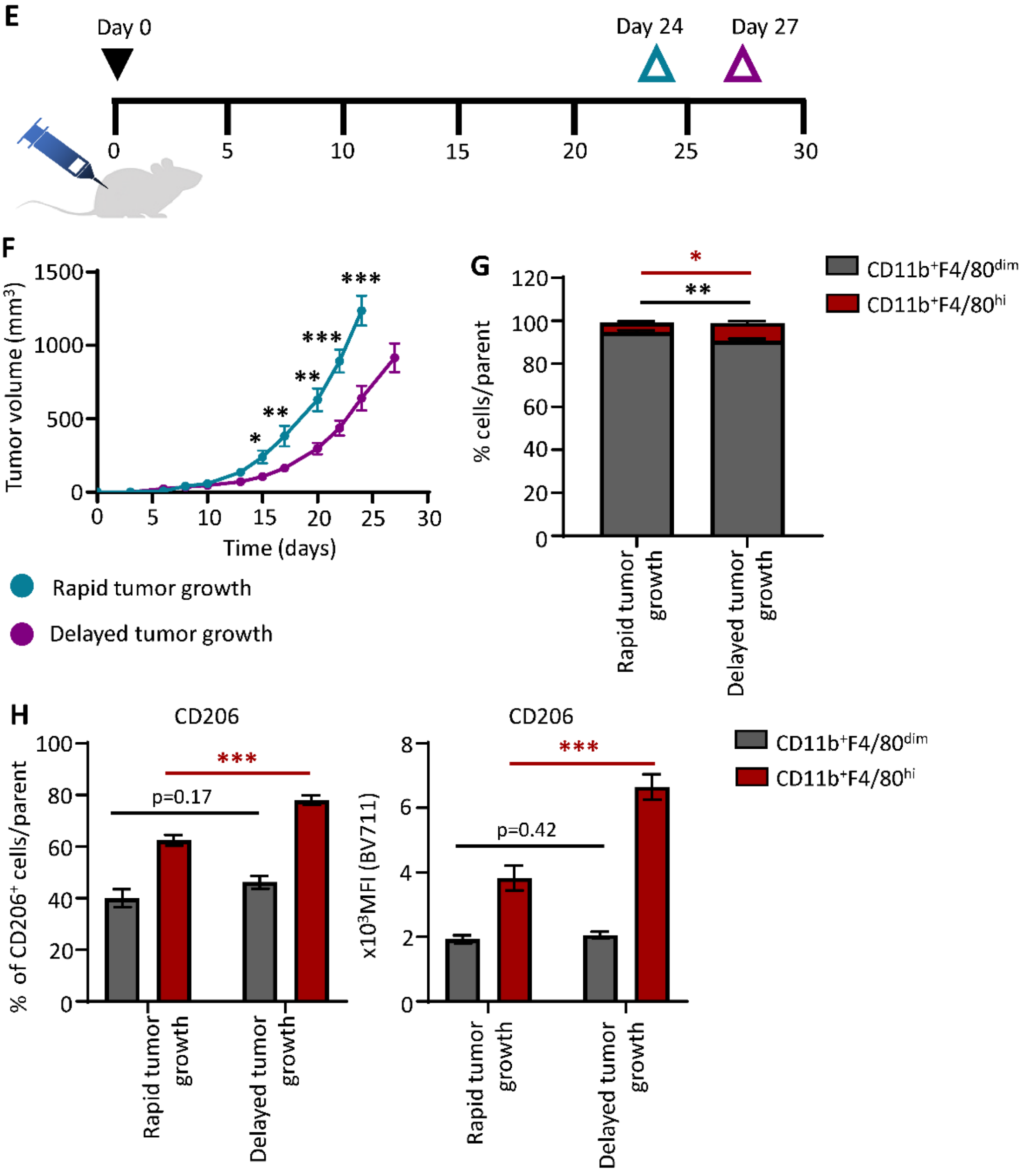

Supplemental Figure S3 Characterization of TAM from B16-F10 melanoma and CT26 colon carcinoma model

(**A**) Representative gating strategy for identification of TAM in B16-F10 and CT26 tumors. TAM were defined as FVS780^{-}, Lin^{-}, CD45^{+}, Ly6C^{-}, Ly6G^{-}, CD11b^{+}, F4/80^{+}. The gates were set according to the respective FMO controls. (**B**) Gating strategy for identifying CD11b^{+}F4/80^{dim} and CD11b^{+}F4/80^{hi} TAM population. Histograms represent expression profile of CD206 on CD11b^{+}F4/80^{dim} (grey histogram) and CD11b^{+}F4/80^{hi} (red histograms) isolated from rapid growth tumors (cyanine blue box,

top row) and delayed growth tumors (purple box, bottom row). Dotted histograms represent respective FMO control for BV711. (**A-B**) Data are representative of 4 mice each from rapid tumor growth and delayed tumor growth group and from two independent experiments. (**C**) Bar diagram represents percentage of $CD206^{+}$ cells with corresponding MFI from $CD11b^{+}F4/80^{dim}$ (grey) $CD11b^{+}F4/80^{hi}$ (maroon) TAM subsets analyzed in rapid and delayed growth tumors by flow cytometry. n=4 mice per group. (**D**) Bar diagrams represent CCL2 levels in the plasma of mice with rapid (cyanine blue) and delayed growth tumor (purple), collected at day 14 post tumor inoculation, compared to tumor naïve mice. n= 4-8 mice per group. **(E)** Timeline of the murine CT26 colon carcinoma tumor model studies, indicating inoculation of CT26 colon carcinoma cells (Day 0) and respective endpoints (Day 24 for rapid growth tumors; Day 27 for delayed growth tumors). (**F)** In vivo CT26 tumor growth curves by two groups identified as rapid growth tumor (cyanine blue) and delayed growth tumor (purple). (**G**) Bar diagram represents percentage of $CD11b^{+}F4/80^{dim}$ (grey) and $CD11b^{+}F4/80^{hi}$ (maroon) TAM analyzed in CT26 rapid and delayed growth tumors by flow cytometry. (**H**) Bar diagrams represent percentage of $CD206^{+}$ cells with corresponding MFI from $CD11b^{+}F4/80^{dim}$ (grey) and $CD11b^{+}F4/80^{hi}$ (maroon) TAM analyzed in CT26 rapid and delayed growth tumors by flow cytometry. (**F-H**) n= 9 mice per group. (**C**-**D; F-H**) Data show mean ± SEM. *$P < 0.05$**$P < 0.01$, *** $P < 0.001$.

Supplemental Figure S4

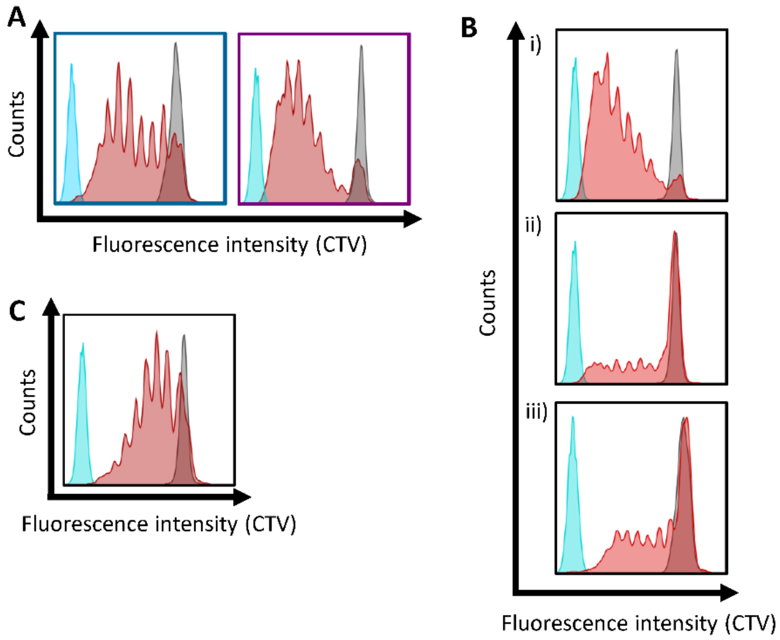

Supplemental Figure S4 Ag cross-presentation by TAM isolated from B16-F10

(**A**) CTV-labelled OT-I CD8a$^+$ T cells were incubated with TAM (CD11b$^+$F4/80$^+$) isolated from B16-F10 rapid growth tumors (cyanine blue box, left) or delayed growth tumors (purple box, right). Red histogram represents proliferation of CTV labelled CD8a$^+$ OT-I T cells co-cultured with OVA-exposed TAM. Data are representative of 4 mice each from rapid tumor growth and delayed tumor growth group and from two independent experiments. (**B**) Red histograms represent proliferation of CTV labelled CD8a$^+$ T cells co-cultured with TAM (CD11b$^+$F4/80$^+$) where TAM were exposed to i) SIINFEKL peptide (positive control) ii) OVA protein (0.1mg/ml) or iii) when TAM were used in 1:10 ratio with CD8a$^+$ OT-I T cells. (**C**) Red histogram represents proliferation of CTV labelled CD8a$^+$ OT-I T cells co-cultured with OVA-exposed CD11b$^+$F4/80^{hi}CD206$^+$ TAM isolated from delayed growth tumors. (**A-C**) Unstimulated unlabeled CD8a$^+$ OT-I T cells (blue histogram) and CTV labelled CD8a$^+$ OT-I T cells co-cultured with BSA-exposed TAM (grey histogram) were used as control.

Supplemental Figure S5

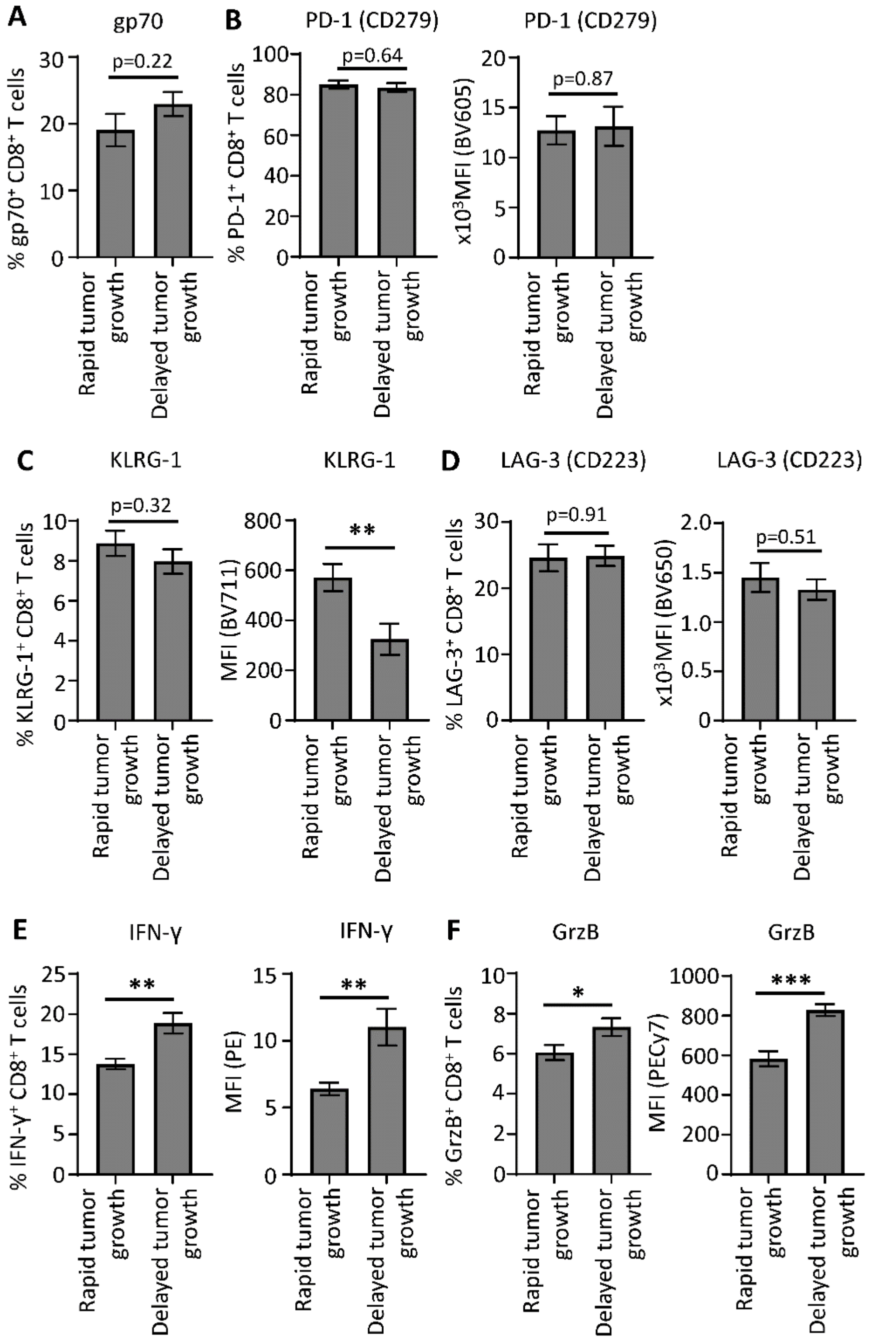

Supplemental Figure S5 Characterization of CD8$^+$ T cells from CT26 colon carcinoma

(**A**) Bar diagram represents percentage of gp70-specific TCR expressing tumor-associated CD8$^+$ T cells in CT26 rapid and delayed growth tumors by flow cytometry. (**B-F**) Bar diagrams show percentage and corresponding MFI of PD-

1^+CD8^+ T cells (**B**), KLRG-1^+CD8^+ T cells (**C**), LAG-3^+CD8^+ T cells (**D**), IFN-γ^+CD8^+ T cells (**E**) and $GrzB^+CD8^+$ T cells (**F**) from CT26 rapid and delayed growth tumors as analyzed by flow cytometry. (**A-F**) Data show mean ± SEM (n=9 mice per group). $^*P < 0.05$, $^{**}P < 0.01$, $^{***}P < 0.001$.

Supplemental Figure S6

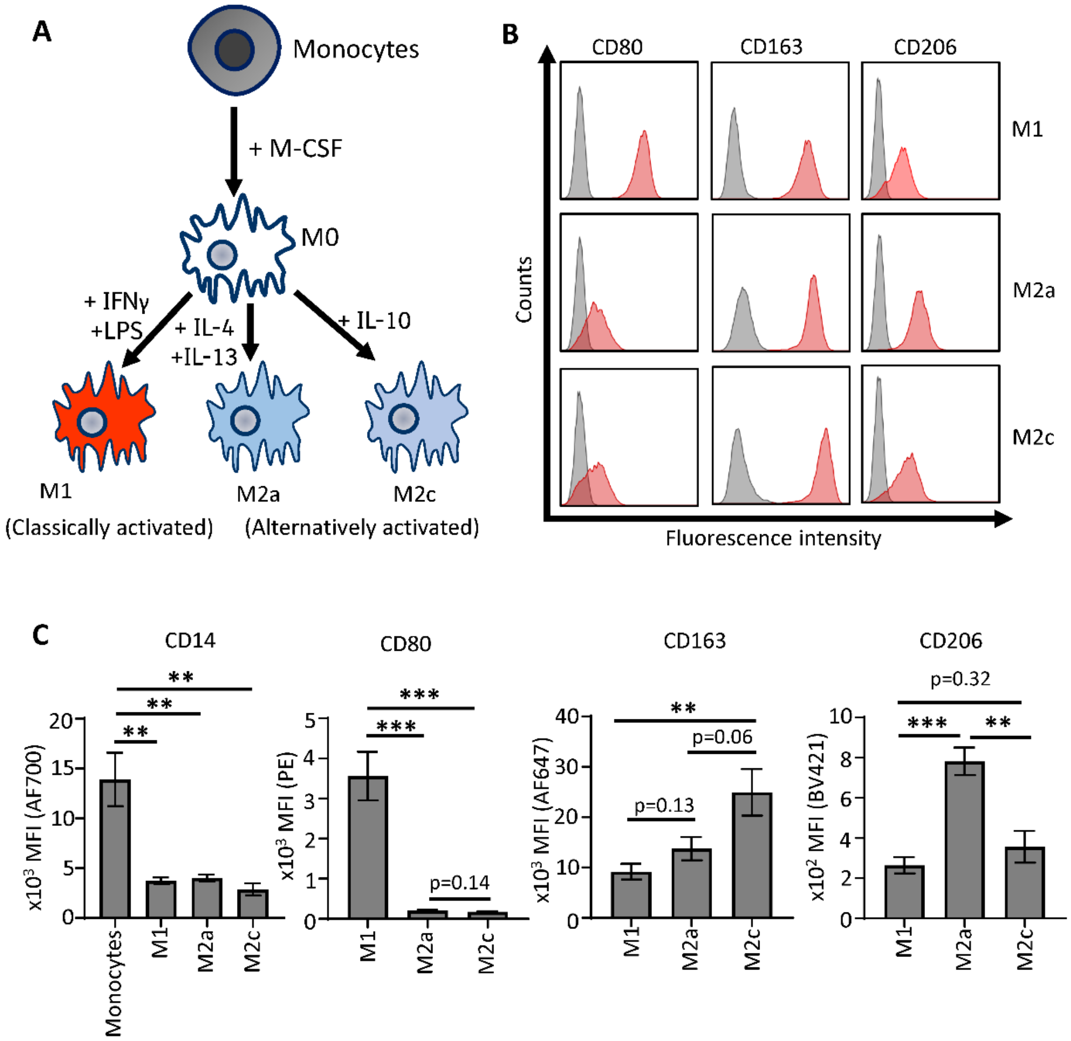

Supplemental Figure S6 Characterization of monocyte-derived macrophage subsets

(**A**) Schematic representation of the differentiation protocol used to obtain human monocyte-derived macrophages (MDM). Isolated $CD14^+$ monocytes were differentiated to classically activated M1 or alternatively activated M2a and M2c macrophages by adding the indicated stimuli. (**B**) At day 7 of differentiation, MDM were analyzed by flow cytometry for the expression of CD80, CD163 and CD206 (red histograms). Grey histograms represent respective control isotype stainings. Data are representative of six different donors. (**C**) Bar diagrams represent MFI of indicated cell

surface markers on MDM subsets as analyzed by flow cytometry. Data show mean ± SEM (n= 6 donors). **$P < 0.01$, ***$P < 0.001$.

Supplemental Figure S7

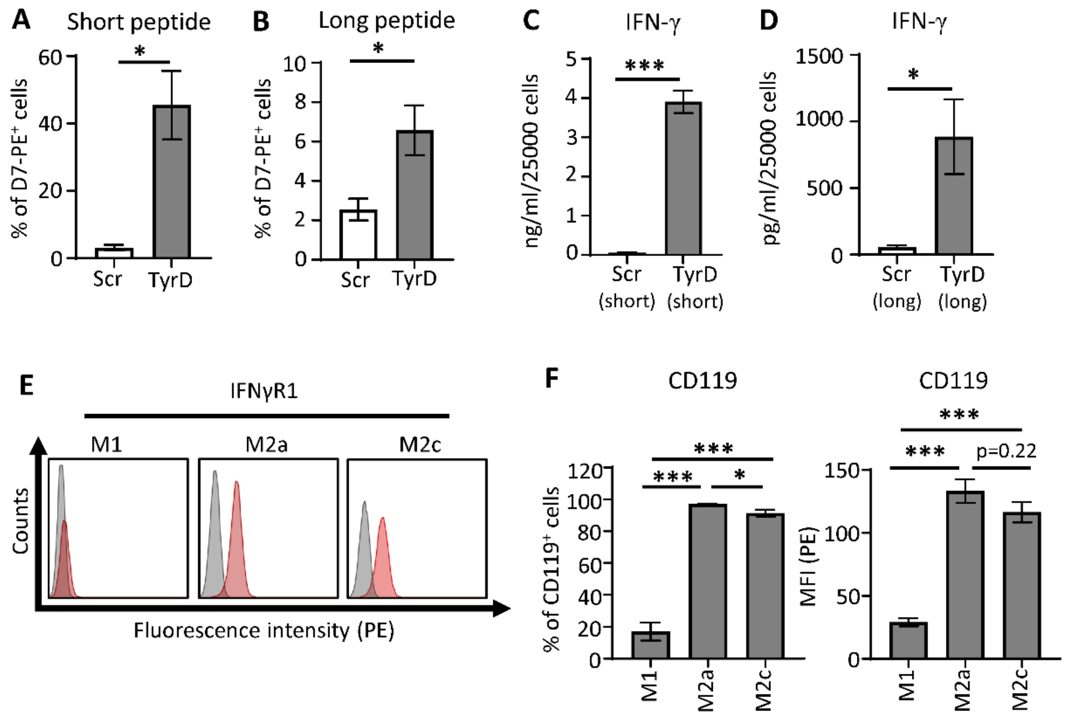

Supplemental Figure S7 Ag cross-presentation by CLEC9A$^+$ cDC1 and expression of CD119 (IFNγR1) on MDM subsets

(**A**) Bar diagram represents percentage of positive cells as detected by staining with D7 Ab upon treatment of CLEC9A$^+$ cDC1 with Scr short peptide (open bars) and TyrD short peptide (filled bars). (**B**) As in (**A**), but CLEC9A$^+$ cDC1 were treated with Scr long peptide (open bars) or TyrD long peptide (filled bars). (**C-D**) Bar diagram represents IFN-γ levels in the T58/cDC1 co-culture supernatant where CLEC9A$^+$ cDC1 were pre-treated with Scr short peptide (open bars) or TyrD short peptide (filled bars) (**C**) and where CLEC9A$^+$ cDC1 were pre-treated with Scr long peptide (open bars) or TyrD long peptide (filled bars) (**D**). (**A-D**) Data show mean ± SEM (n= 3 donors). (**E**) Expression profile of CD119 (IFNγR1) on M1, M2a and M2c MDM subsets (red histograms). Grey histograms represent control isotype staining. (**F**) Bar diagrams show percentage of CD119$^+$ cells with corresponding MFI as analyzed by flow cytometry. Data show mean ± SEM (n= 4 donors). (**A-D** and **F**) *$P < 0.05$, ***$P < 0.001$.

Supplemental Figure S8

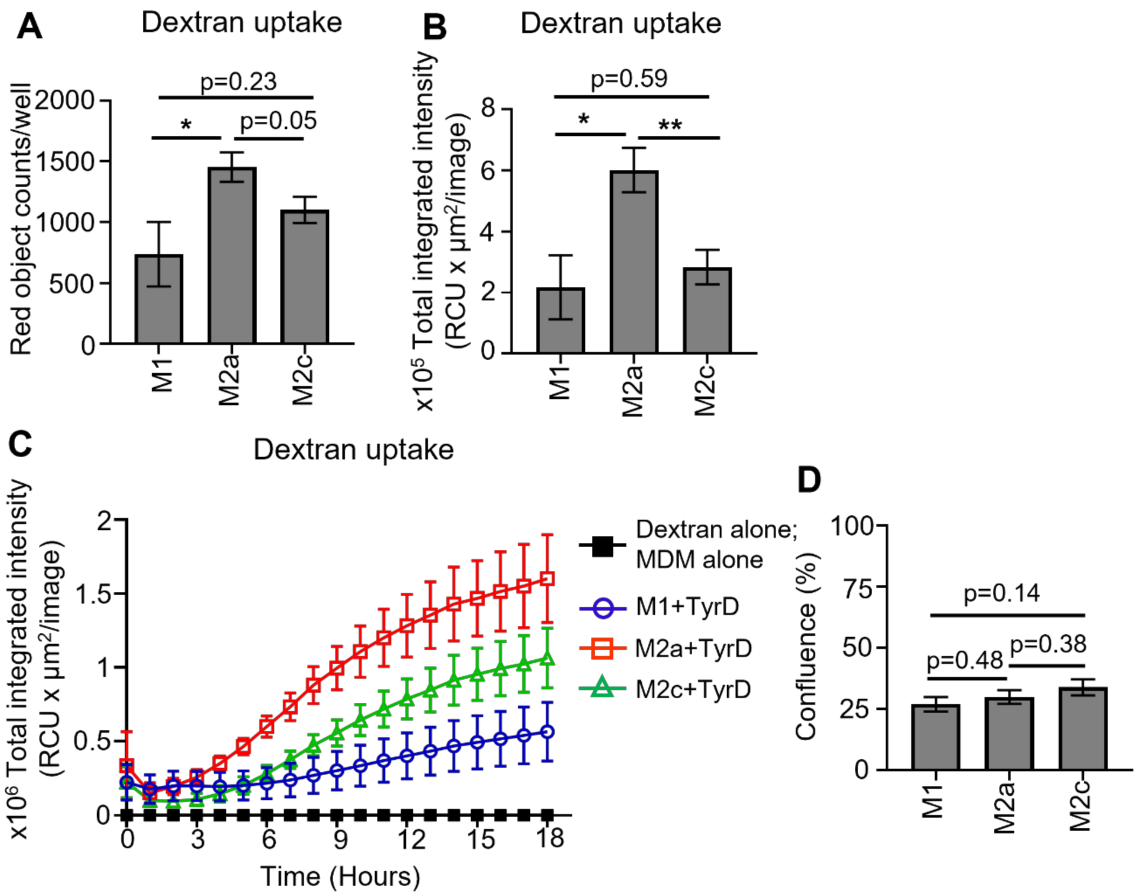

Supplemental Figure S8 Endocytosis of pHrodo™ labelled dextran by MDM subsets

(**A-C**) Differentiated human MDM were co-incubated with pHrodo™ red labelled Dextran. Ag uptake was analyzed using Incucyte® S3 live cell analysis system. Bar diagrams represent number of red objects/well (**A**) and total integrated intensity (**B**) as analyzed using Incucyte® software at 6 h. (**C**) M1 (blue circles), M2a (red squares) and M2c (green triangle) MDM were co-incubated with pHrodo™ red labelled dextran. Ag uptake was recorded over 18 h. Graphs also show baseline values for dextran alone and MDM subsets alone (black squares). Data show mean ± SEM (n= 6 donors). *$P < 0.05$, **$P < 0.01$. (**D**) Bar diagram representing percentage confluence of seeded MDM as assessed at 0 h. Data show mean ± SEM (n= 8 donors).

Supplemental Figure S9

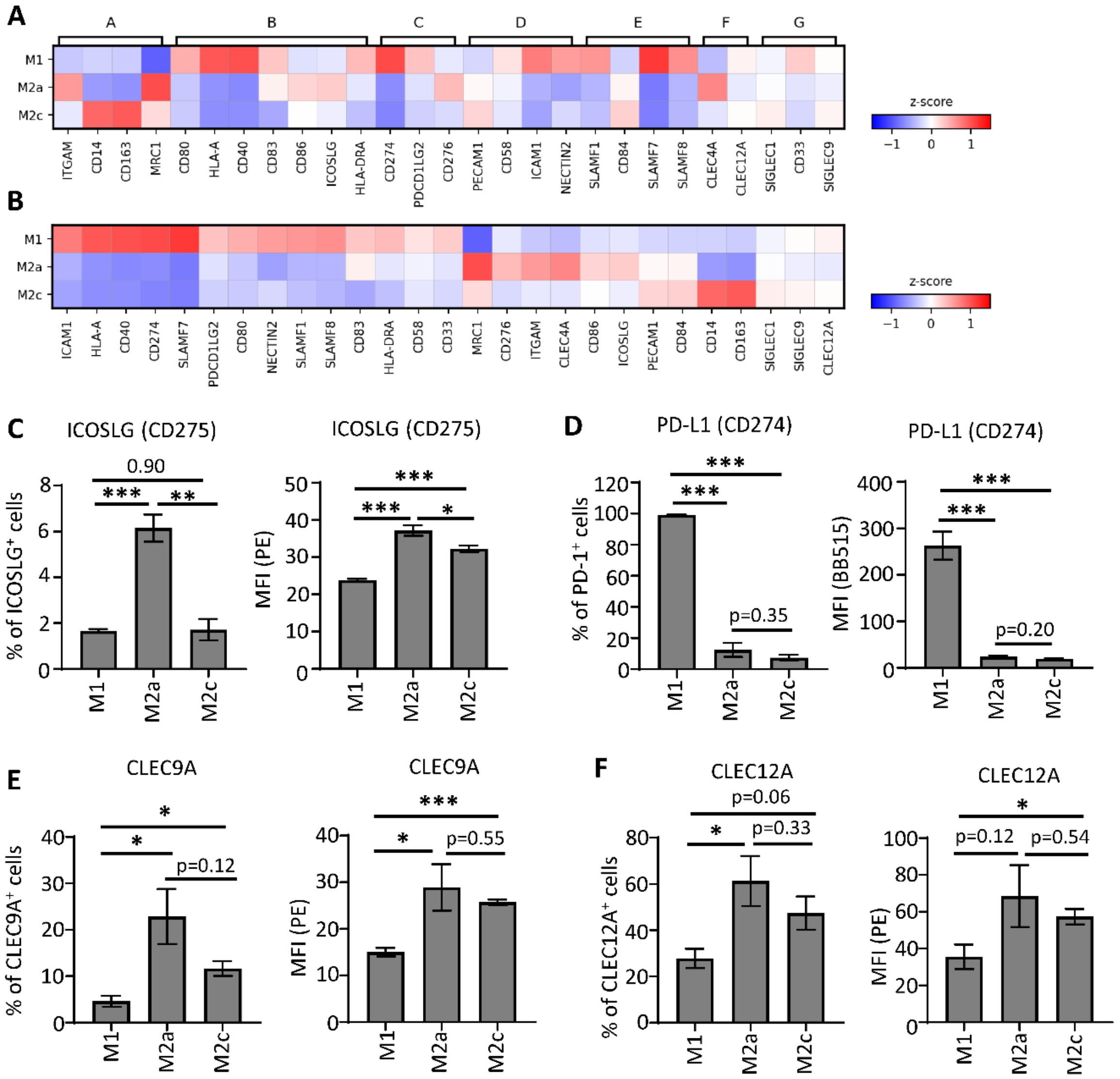

Supplemental Figure S9 CD206⁺ macrophages express high levels of costimulatory receptors CD86 and ICOSLG but low levels of inhibitory receptor CD274

(**A**) Heat map showing mean z-score of each analyzed cell subset in the corresponding MDM group. Genes are grouped by as follows: 'Group A': Macrophage markers; 'Group B': Receptors involved in T cell co-stimulation; 'Group C': Receptors involved in T cell inhibition; 'Group D': Receptors involved in cell adhesion/migration/synapse formation; 'Group E': Receptors of SLAM family; 'Group F': Receptors of C-type lectin family; 'Group G': Receptors of Siglec family. Columns represent cumulative z-score

of analyzed individual cells from respective MDM subsets. Color scheme is based on z-score distribution. Minimum z-score -1 (blue) to maximum z-score 1 (red). (**B**) Data as in (**A**) but genes are ordered based on their specific expression in M1, M2a and M2c MDM subsets. (**C-F**) Bar diagrams show percentage of ICOSLG^{+} (**C**) PD-L1^{+} (**D**), CLEC9A^{+} (**E**) and CLEC12A^{+} (**F**) cells along with corresponding MFI of as analyzed by flow cytometry. Data show mean ± SEM (n= 4 donors). **P* < 0.05, ***P* < 0.01, ****P* < 0.001.

Supplemental Figure S10

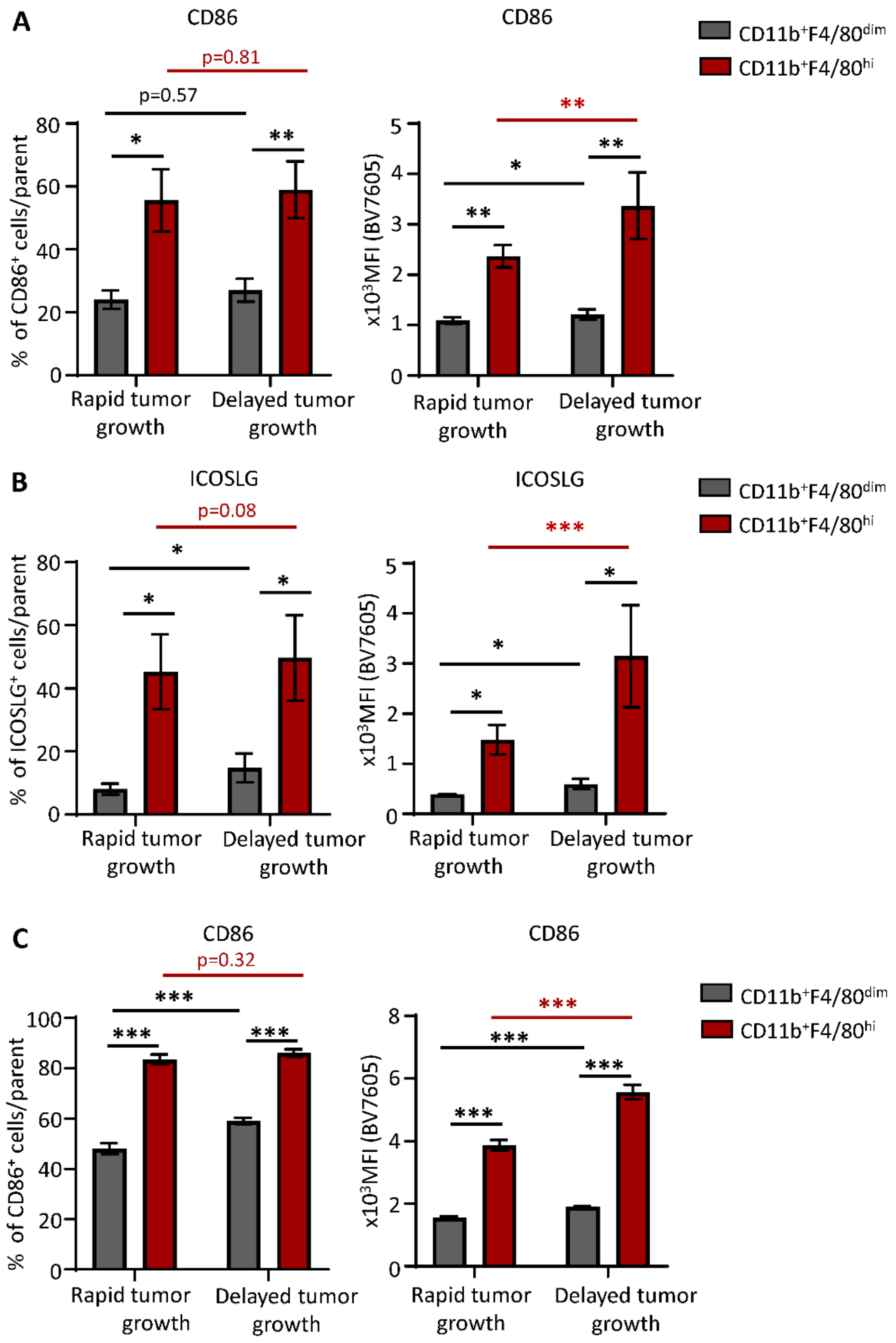

Supplemental Figure S10 (continued)

D

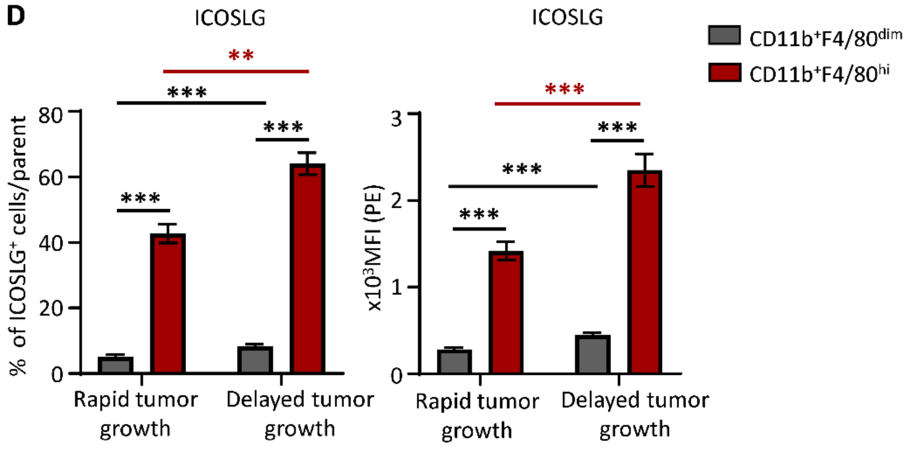

Supplemental Figure S10 Analysis of CD86 and ICOSLG expression on TAM isolated from B16-F10 and CT26 tumor models

(**A-B**) Bar diagrams represent percentage of $CD86^+$ cells (**A**) and $ICOSLG^+$ cells (**B**) with corresponding MFI from $CD11b^+F4/80^{dim}$ (grey) and $CD11b^+F4/80^{hi}$ (maroon) TAM analyzed in B16-F10 rapid and delayed growth tumors by flow cytometry. Data show mean ± SEM (n= 5-7 mice per group). (**C-D**) Bar diagram represents percentage of $CD86^+$ cells (**C**) and $ICOSLG^+$ cells (**D**) with corresponding MFI from $CD11b^+F4/80^{dim}$ (grey) and $CD11b^+F4/80^{hi}$ (maroon) TAM analyzed in CT26 rapid and delayed growth tumors by flow cytometry. Data show mean ± SEM (n= 9 mice per group). (**A-D**) **P* < 0.05, ***P* < 0.01, ****P* < 0.001.

Supplemental Figure S11

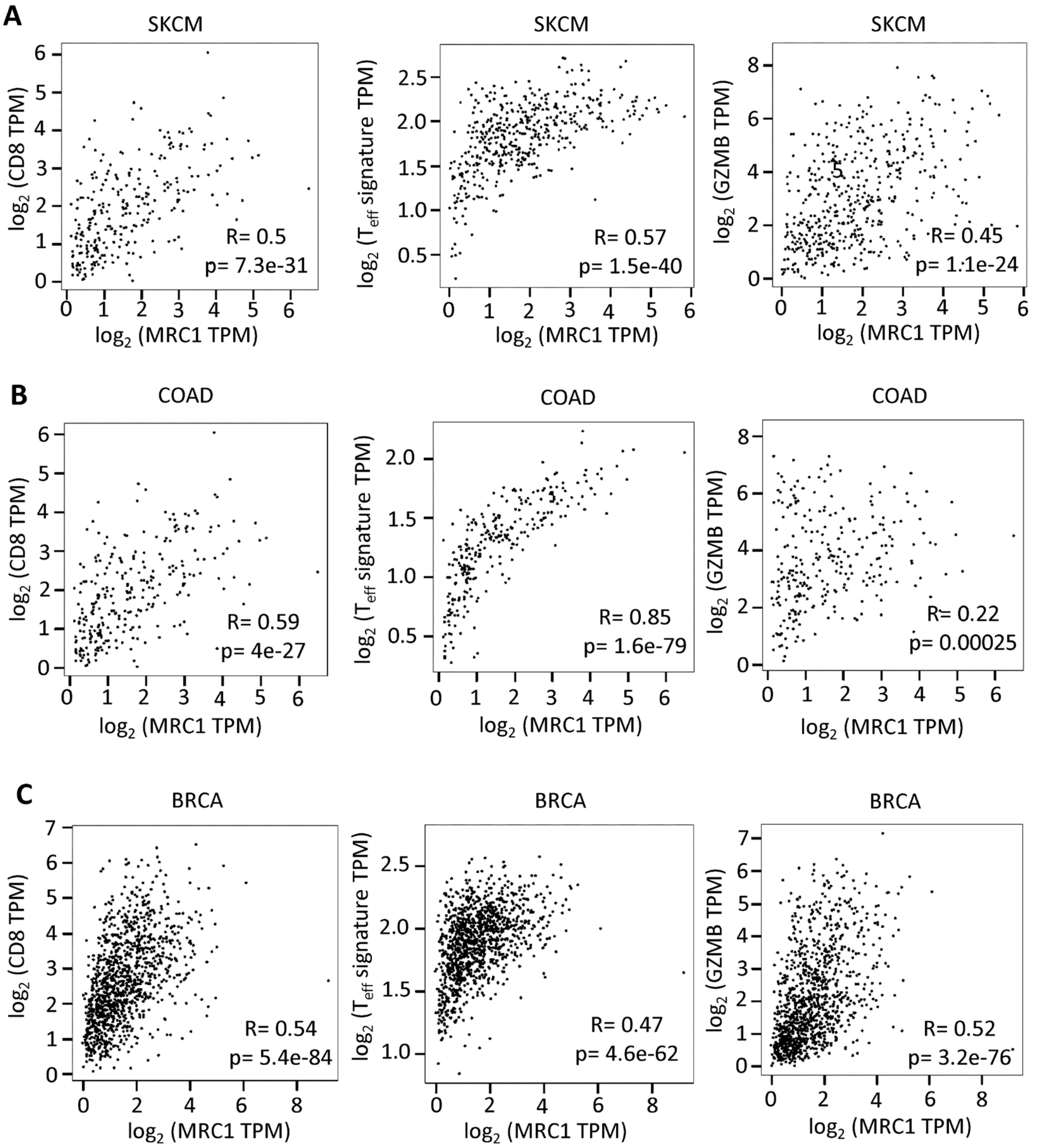

Supplemental Figure S11 Co-relation analysis of *MRC1* (CD206) expression CD8$^+$ T cell signature in cancer patients

(**A-C**) Correlation analysis performed between *MRC1* mRNA and *CD8* mRNA, between *MRC1* and effector T cell gene signature (*CX3CR1*, *FGFBP2*, *FCGR3A*), as well as between *MRC1* mRNA expression and *GZMB* mRNA in skin cutaneous melanoma patients (n=461) (**A**), in colon adenocarcinoma patients (n=275) (**B**), and in breast invasive carcinoma patients (n=1085) (**C**) from TCGA database. The analysis was performed using GEPIA web-based tool.

Supplemental Tables

Supplemental Table 1: List of mouse antibodies used for flow cytometry analysis

Target	Clone	Fluorochrome	Catalog No.	Provider
mCD90.2	30-H12	PECy7	105326	Biolegend
mTCRgd	GL3	PECy7	118124	Biolegend
mCD19	6D5	PECy7	115520	Biolegend
mNK1.1	PK136	PECy7	108714	Biolegend
mCD45	30-F11	APC	559864	BD Biosciences
mCD11b	M1/70	BV421	562605	BD Biosciences
mF4/80	BM8	BV605	123133	Biolegend
mLy6C/Ly6G	RB6-8C5	AF488	108417	Biolegend
mCD206	C068C2	BV711	141727	Biolegend
mCD86	GL-1	BV 605	105037	Biolegend
mCD275	HK5.3	PE	107405	Biolegend
mCD3e	145-2C11	AF488	100321	Biolegend
mCD4	GK1.5	APC/Cy7	100414	Biolegend
mCD8	53-6.7	PerCP/Cy5.5	100734	Biolegend
mCD279	29F.1A12	BV 605	135220	Biolegend
mCD223	C9B7W	BV650	125227	Biolegend
mKLRG-1	2F1/KLRG1	BV711	138427	Biolegend
Rat IgG1, κ	RTK2071	PE	400408	Biolegend
mIFN-γ	XMG1.2	PE	505808	Biolegend
Rat IgG2a, κ	RTK2758	PE-Cy7	400522	Biolegend
mGranzyme B	NGZB	PE-Cy7	25-8898-82	Invitrogen

Supplemental Table 2: List of human antibodies used for flow cytometry analysis

Target	Clone	Fluorochrome	Catalog No.	Provider
hCD14	M5E2	AF700	557923	BD Biosciences
hCD80	L307.4	PE	557227	BD Biosciences
hCD163	GHI/61	AF647	562669	BD Biosciences
hCD206	19.2	BV421	566281	BD Biosciences
HLA-A02	BB7.2	APC	561341	BD Biosciences
hCD40	5C3	BB515	565927	BD Biosciences
hCD274	MIH1	BB515	564554	BD Biosciences
HLA-DR	G46-6	APC	559866	BD Biosciences
hCD86	2331	PE	555658	BD Biosciences
hCLEC4A	I3-612	AF647	558220	BD Biosciences
hCLEC9A	3A4	PE	563488	BD Biosciences
hCLEC12A	50C1	PE	562566	BD Biosciences
CD119	GIR-208	PE	558934	BD Biosciences
hICOSLG	2D3/B7-H2	AF647	564278	BD Biosciences
hCD8	RPA-T8	BV421	562428	BD Biosciences
hCD25	BC96	BB5	567318	BD Biosciences

Supplemental Table 3: Lists of tetramers used for flow cytometry analysis

Target protein	Fluorochrome	Target peptide	Order No.	Provider
CMV	PE	NLVPMVATV	TB-0010-1	MBL International
Ovalbumin	PE	SIINFEKL	TB-5001-1	MBL International
Gp70	PE	SPSYVYHQF	TB-M521-1	MBL International

Supplemental methods

Generation of TCR like D7 antibody

The sequence of the $TyrD_{369-377}$/HLA-A02 complex specific murine IgG2a Ab clone D7 was described previously (16). The Ab DNA sequence was synthesized (Geneart) and cloned in the pTT5 expression vector (National Research Council, Canada). The Ab was expressed in CHO-3E7 cells (National Research Council, Canada) after transient transfection using Trans IT PRO transfection reagent (Mirus Bio). After cultivation for 10 days, culture supernatant was clarified using G4 filter (Sartopore). The Ab was purified from supernatant by protein A affinity chromatography using Mab select SuRe resin (GE Healthcare) and subsequent size-exclusion chromatography using a Superdex 200, Hi load 26/600, 320 mL column (GE Healthcare). Eventually, the protein was formulated in 50 mM sodium acetate, 100 mM NaCl, pH 5.0, and characterized by SDS-PAGE, analytical size-exclusion chromatography (Agilent PL1580-5301 column) coupled to multi-angle light scattering, and mass spectrometry. Analytical characterization by intact mass spectrometry, SDS-PAGE and size-exclusion chromatography showed the purified D7 Ab showed expected molecular weight with >99% purity and <1% aggregated species (data not shown).

Culturing of COLO829, HCT116 and T2 cell lines

$Tyrosinase^{+}HLA\text{-}A02^{-}$ melanoma cell line COLO829, $HLA\text{-}A02^{+}TAP^{-}$ T2 cell line and colon carcinoma HCT116 cell line, mouse melanoma B16-F10 cell line and colon carcinoma CT26.WT cell line were purchased from American tissue Culture Collection (ATCC). COLO829 cells were cultured in RPMI1640 supplemented with 10% heat-inactivated FCS (Gibco), 1 x Glutamax (Gibco), 1 x Sodium Pyruvate (Gibco) and 1 x MEM non-essential amino acids (Gibco). T2 cells were cultured in IMDM supplemented with 1 x Glutamax (Gibco) and 20% heat-inactivated FCS (Gibco).

HCT116 cells were cultured in McCoy's 5A Medium supplemented with heat-inactivated 10 % FCS (Gibco). B16-F10 or B16-F10-OVA cells were cultured in DMEM supplemented with heat-inactivated 10% FCS (Gibco). CT26.WT cells were cultured in RPMI1640 supplemented with heat-inactivated 10% FCS (Gibco).
